# The Trilineage Coexistence Observed During the Differentiation of Porcine EPSCs

**DOI:** 10.3390/cells15100954

**Published:** 2026-05-21

**Authors:** Lihua Zhao, Yanglin Chen, Xiyun Guo, Meng Zhou, Tianxu Guo, Junjun Ma, Manling Zhang, Linxin Cheng, Jinbo Yu, Yu Zhang, Guang Yang, Rongfeng Li, Xihe Li

**Affiliations:** 1 The State Key Laboratory of Reproductive Regulation and Breeding of Grassland Livestock, College of Life Science, Inner Mongolia University, Hohhot 010020, China; zhaolihua@njmu.edu.cn (L.Z.);; 2 Jiangsu Provincial Key Laboratory of Biological Therapy for Organ Failure, Nanjing Medical University, Nanjing 211166, China; 3 National Center of Technology Innovation for Dairy, Hohhot 010020, China; 4 Key Laboratory of Targeted Intervention of Cardiovascular Disease, Collaborative Innovation Center for Cardiovascular Disease Translational Medicine, Nanjing Medical University, Nanjing 211166, China; 5 Inner Mongolia Saikexing Institute of Breeding and Reproductive Biotechnology in Domestic Animal, Hohhot 011517, China

**Keywords:** porcine, EPSCs, differentiation, trilineage, coexistence

## Abstract

The mammalian early embryo possesses totipotency and can be captured as extended pluripotent stem cells (EPSCs). The first two cell differentiations result in epiblast, primitive endoderm, and trophectoderm, with the trilineage coexisting in a unified uterine microenvironment. Nevertheless, the in vitro counterparts—primed PSCs, trophoblast stem cells (TSCs), and extraembryonic endoderm (XEN) cells—require a distinct culture system. In this study, we successfully derived stable porcine EPSCs from fibroblasts at 35% efficiency, and interestingly observed that these EPSCs differentiated in parallel and gave rise to the transient coexistence (36–84 h) of trilineage cells when cultured in a single system (LCDM: hLIF, CHIR99021, DiM, and MiH). Then, XEN cells gradually predominated and eventually became the sole population in prolonged LCDM culture. However, TSCs and primed PSCs had to be differentiated from EPSCs under their respective culture system. EPSCs can differentiate into PGC-like cells independently of genetic modification and contribute to mouse neurula-stage embryos. Collectively, the trilineage coexistence phenomenon may provide novel insight into an early embryogenesis mechanism and strategy for porcine blastoid construction.

## 1. Introduction

After two lineage segregation events, the mammalian conceptus organizes into three distinct lineages within the blastocyst: epiblast (EPI), primitive endoderm (or hypoblast, HYPO), and trophectoderm (TE) [1]. Among in vitro-maintained pluripotent stem cells (PSCs), extended pluripotent stem cells (EPSCs) demonstrate the broadest developmental capacity—differentiating into all three blastocyst lineages—with a plasticity resembling totipotent early blastomeres before lineage commitment.

In 2017, Liu [2] and Deng [3] groups independently established mouse EPSCs with pluripotency features of 4–8-cell embryos. Gao et al. later extended this pluripotent state across species, deriving both porcine and human EPSCs through small-molecule optimization adapted from mouse systems [4]. Yoshimatsu et al. further showed that marmoset EPSC-like cells integrate into mouse interspecies chimeras [5]. Collectively, these studies position EPSCs as a powerful platform for developmental modeling and uterine microenvironment research, with derivations possible from embryos, embryonic stem cells (ESCs), and induced PSCs (iPSCs).

The three blastocyst lineages can be stably captured in vitro as primed PSCs, extraembryonic endoderm (XEN) cells, and trophoblast stem cells (TSCs) under appropriate signaling environments. This correspondence between in vitro populations and in vivo lineages suggests unique uterine microenvironments coordinate self-renewal and differentiation during the pre- to post-implantation transition [6,7]. Faithfully recapitulating these niche signals during EPSC differentiation is critical for deciphering early developmental mechanisms and advancing applications in organoid and chimera research [6,8].

The indefinite in vitro self-renewal capacity of PSCs parallels evolutionarily conserved delayed embryonic development [9]. For example, mTOR inhibition alone prolongs the blastocyst stage in mice and humans for weeks, mirroring sustained pluripotency and differentiation potential in stem cells under similar conditions [10]. Beyond mTOR, multiple signaling pathways regulate stem cell states [11,12], and targeted pathway modulation enables blastoid generation (stem cell-derived structures recapitulating early embryogenesis) [13,14,15]. However, existing models show limited progression from blastoid to gastruloid stages—challenges exacerbated in large animals [16,17], where models often exhibit incomplete lineage representation and developmental arrest prior to gastrulation [18,19]. Overcoming these limitations requires precise recapitulation of the transition from pluripotency to lineage commitment, which may yield mechanistic insights into developmental arrest disorders.

A fundamental characteristic of multicellular organisms is generating lineage-specific cells while maintaining plasticity for stem cell state interconversion [20]. Signaling networks regulating pluripotency and lineage specification (e.g., LIF, WNT, MAPK, ERK) exhibit extensive crosstalk and pleiotropy [21,22,23]. While conventional culture enables individual lineage-specific stem cells to be derived [24,25,26], evidence for the simultaneous emergence of multipotent progenitors giving rise to trilineage stem cells remains limited. In this study, we generated pEPSCs from porcine embryonic fibroblasts (PEFs) using a modified induction protocol. These cells exhibit typical EPSC morphology, express core pluripotency markers, possess transcriptomic profiles consistent with extended pluripotency, differentiate into three germ layers, and contribute to xenogeneic chimeras in mouse neurula-stage embryos. When these pEPSCs were cultured in LCDM medium (hLIF, CHIR99021, DIM, and MIH), we observed the transient coexistence of trilineage stem cells (PSCs, TSCs, and XEN cells), replicating aspects of early embryonic lineage segregation. Under prolonged LCDM culture, the XEN cells eventually gave rise to XEN cells. Notably, pEPSCs also differentiated into TSCs, primed PSCs, and primordial germ cell-like cells (PGCLCs) without genetic modification. These findings establish the LCDM system as a valuable tool for investigating porcine embryogenesis and improving in vitro developmental models, while providing a strategy for porcine blastoid construction.

## 2. Materials and Methods

### 2.1. Generation of pEPSCs

PEFs were isolated from the offspring of female Fragrance pig × male wild boar and cultured in DMEM (Invitrogen, Waltham, MA, USA) supplemented with 15% FBS (BI, Kibbutz Beit Haemek, Israel). PEFs (80% confluence) were digested, and eight exogenous transcription factors (p5F + h3F) were transfected into them using the Basic Nucleofector Kit for Primary Mammalian Fibroblasts (Lonza, Basel, Switzerland). The transfection mixture included PB-Tre-pOSKM (including porcine *Oct4*, *Sox2*, *Klf4*, and *c-Myc*), PB-Tre-pNhL (including porcine *Nanog* and human *LIN28*), PB-Tre-hRL (including human *RARG* and *LRH1*, which is also known as *NR5A2*), PB-EF1α-transposase, and PB-CCAG-rtTA [4]. These transfected PEFs were seeded onto STO feeders in M15 medium consisting of knockout DMEM, 15% FBS, 1% Glu-PS, 1% NEAA, 0.1 mM β-mercaptoethanol (all Invitrogen), 50 µg/mL Vitamin C (VC, Sigma, Darmstadt, Germany), 10 ng/mL hLIF (Millipore, Saint Louis, MO, USA), and 1 mg/mL Dox (Tocris, Bristol, UK). Emerging colonies were picked, dissociated with TrypLE (Invitrogen), and passaged onto STO feeders.

After 1–2 days in M15, the cells were transferred into PEPSC medium [27] consisting of Knockout DMEM, 1% N2, 1% B27, 1% Glu-PS, 1% NEAA, 0.1 mM β-mercaptoethanol, 0.2 µM CHIR99021 (Tocris), 0.3 µM WH-4-023 (Selleck, Houston, TX, USA), 2.5 µM XAV939 (Sigma), 65.0 µg/mL VC, 10.0 ng/mL hLIF, 20 ng/mL Activin A (PeproTech, Cranbury, NJ, USA), and 0.3% FBS. When the cells were passaged onto Geltrex-coated (1% in DMEM/F12, Invitrogen) plates, the FBS concentration was increased to 3%.

### 2.2. Embryoid Bodies (EBs) Formation Assay

pEPSCs were dissociated and separated from STO feeder cells by gelatin pre-plating and then cultured in hanging drops (PEPSC medium without hLIF/small molecule) for 7 days. EBs were transferred to gelatin-coated plates and cultured for 5–7 more days, and three-germ-layer differentiation was analyzed by RT-qPCR and immunofluorescence.

### 2.3. Chimeric Embryo Generation

ICR mice were obtained from the Animal Core Facility of Nanjing Medical University and housed in a barrier facility with a 12 h on/12 h off light cycle. All mouse work was performed with the approval of the Institutional Animal Care and Use Committee of Nanjing Medical University (IACUC-2112051), and the methods were carried out following the animal ethical guidelines of the Nanjing Medical University.

Female ICR mice were superovulated with 5 IU of Pregnant Mare Serum Go-nadotropin (PMSG) and 5 IU of human chorionic gonadotropin (hCG) 48 h apart and mated overnight with male mice. The pregnant mice were euthanized and dissected on embryonic day 7.5 (E7.5), and E7.5 ICR mouse embryos were collected via uterine flushing. GFP-pEPSCs were generated by transfecting a CAG-GFP vector and manual picking; about ~15 of these cells were injected into the middle post-posterior of each E7.5 mouse embryo. Injected embryos were cultured in PEPSC medium supplemented with 12.5% KSOM (Millipore) and 12.5% human serum (37 °C, 5% CO_2_, 12–24 h) and imaged using a Nikon Ti2 microscope.

### 2.4. HYPO-Lineage Differentiation

From the Geltrex-coated plates, pEPSCs were seeded onto 1% gelatin-coated plates in LCDM medium consisting of knockout DMEM, 0.1 mM β-mercaptoethanol, 1% NEAA, 1% PS, 1% Glutamax, 10% knockout serum replacement (KOSR), 10% FBS, 10 ng/mL hLIF,1 µM CHIR99021, 2 µM DIM (Tocris), 2 µM MIH (Tocris), and 2 µM Y27632 (Tocris). They were passaged every 5–6 days using Accutase.

### 2.5. TE-Lineage Differentiation

After being cultured on Geltrex-coated plates for 1~2 passages, pEPSCs were cultured in LCDM medium for 24, 48, or 72 h. Afterwards, the medium was switched to porcine TSC consisting of knockout DMEM, 2.5% FBS, 1% NEAA, 0.1 mM β-mercaptoethanol, 1% Glu-PS, 3 µM CHIR99021, 65.0 µg/mL VC, 2.5 ng/mL hLIF, 125 µg/mL bovine serum albumin (BSA; Sigma), 1 µM SB431542 (Selleck), 1% ITS-X supplement, 50 ng/mL hEGF, 1 µM A83-01 (Sigma), and 5 µM Y27632. The cells were passaged every 3–4 days using Accutase (Invitrogen).

### 2.6. EPI-Lineage Differentiation

pEPSCs were seeded onto STO feeders, and the medium was switched to porcine primed ESC [28,29] consisting of α-MEM, 20% KOSR, 1% Glutamax, 1% NEAA, 55 µM β-mercaptoethanol, 1% PS, 1% ITS, 20 ng/mL human EGF (hEGF; all Invitrogen), 10 ng/mL Activin A, and 20 ng/mL bFGF (PeproTech). Colonies were passaged every 4–5 days using 1 IU/mL Dispase (STEMCELL, Vancouver, BC, Canada).

### 2.7. PGCLC Induction

pEPSCs were separated from the STO feeders by gelatin pre-plating. These cells were then seeded onto fibronectin-coated plates and cultured for 24–48 h in PEPSC medium supplemented with 50 ng/mL Activin A, 3 µM CHIR99021, and 10 μM Y27632 to iMeLCs. Then, iMeLCs were dissociated and cultured in U-shaped 96-well plants (Corning, NY, USA) at a density of 5000 cells per well in PGCLC induction medium [PEPSC medium supplemented with 10 ng/mL hLIF, 50 ng/mL hEGF, 0.2 µg/mL BMP4 (PeproTech) and 0.1 µg/mL SCF (PeproTech)] for 5–6 days.

### 2.8. Karyotype Analysis

Cells (60–70% confluence) were incubated with 0.01 µg/mL colcemid (BI) at 38.5 °C for 2 h, and single-cell suspensions were prepared via preheated 0.56% KC1 (38.5 °C, 30 min), and fixed in ice-cold methanol: glacial acetic acid (3:1, *v*/*v*; 3×, room temperature). Cell pellets were then resuspended in fresh fixative, dropped onto pre-chilled slides, stained with Giemsa (Sigma) for 20 min, and examined. At least 30 metaphase spreads were analyzed per experiment.

### 2.9. Alkaline Phosphatase (AP) Staining

Cells (70–90% confluence) were washed with PBS and fixed with 4% paraformaldehyde for 10 min at room temperature. AP activity was detected using the BCIP/NBT Substrate (Promega, Fitchburg, WI, USA) per the manufacturer’s protocol, and these cells were imaged using a Nikon Ti2 microscope (Nikon, Tokyo, Japan).

### 2.10. Immunofluorescence Analysis

Cells were washed with PBS, fixed with 4% paraformaldehyde (for 10 min), permeabilized with 1% TritonX-100 in PBS (for 1 h), and blocked with 5% BSA in 0.1% TritonX-100 (for 1 h). Primary antibodies were incubated overnight at 4 °C, and after 3 washes with 0.1% TritonX-100 (for 5 min each), species-appropriate secondary antibodies were incubated for 1 h (at room temperature). Nuclei were counterstained with DAPI (Sigma, 5 min). Images were acquired using a Nikon Ti2 microscope and densitometry was performed using Image J (Java 1.8.0, National Institutes of Health, USA). Antibody details and their corresponding lineages are shown in Table S1.

### 2.11. Western Blot Analysis

Cells were extracted in RIPA buffer (ice, 20 min). Lysates were centrifuged (14,000× *g*, 15 min) and the resulting supernatants were quantified using the Pierce^TM^ BCA Kit (Thermo Fisher Scientific, Waltham, MA, USA). Equal protein amounts were separated by 10% SDS-PAGE gels and transferred to polyvinylidene fluoride (PVDF) membranes using a semi-dry transfer system (Bio-Rad, Hercules, CA, USA). These membranes were blocked with 5% non-fat milk in /TBST (for 1 h at room temperature) and incubated with primary antibodies overnight at 4 °C. After 3 TBST washes, secondary antibodies were incubated for 2 h at room temperature. Bands were visualized using a chemiluminescence (ECL) substrate and imaged with the ChemiDoc XRS+ system (Bio-Rad). β-ACTIN was used as the loading control and band intensities were quantified via Image J software. Antibody details and their corresponding lineages are shown in Table S2.

### 2.12. Quantitative Real-Time PCR (RT-qPCR) Analysis

Total RNA was extracted using the Total RNA Extraction Kit (Promega), and 1 µg of this was used to synthesize cDNA using the HiScriptII Q RT SuperMix (Vazyme, Nanjing, China). RT-qPCR was performed on the 7500 Real-time System (Applied Biosystems, Waltham, MA, USA) with the ChamQ SYBR qPCR Master Mix (Vazyme). Relative expression was calculated using the 2^−ΔΔCt^ method (Gapdh as internal control), and the primer sequences and their corresponding lineages are listed in Table S3.

### 2.13. RNA Sequencing and Transcriptome Analysis

pEPSCs were dissociated and lysed in RNAiso Plus reagent (Takara, Kusatsu, Japan) at a density of 5 × 10^6^ cells/mL and RNA integrity was assessed using an Agilent 2100 Bioanalyzer (Agilent Technologies, Inc. Beijing, China). Libraries were prepared using the VAHTS Universal V6 RNA-seq Kit and sequenced on an Illumina Novaseq 6000 (150 bp paired-end reads; OE Biotech, Shanghai, China). Gene expression was quantified as FPKM and read counts were quantified by HTSeq-count. PCA was performed using R (v 3.2.0) and edge R (v 1.22.2, *p* < 0.05, foldchange > 2) was used to carry out differential expression analysis.

RNA-seq data were deposited in GitHub (https://github.com/Zhao1477/sequencing, accessed on 21 March 2024). Data for porcine EPSC-GXF were obtained from GitHub (https://github.com/dbrg77/pig_and_human_EPSC, accessed on 21 March 2024) [4], those for porcine naïve and primed ESCs were downloaded from GSE183270 [25], those for porcine EPI and HYPO lineages were downloaded from GSE112380 [30], and those for porcine ICM and TE lineages were downloaded from GSE139512 [31].

### 2.14. Single-Cell RNA Sequencing Analysis

pEPSCs were seeded onto Geltrex-coated 6-well plates and cultured in LCDM medium for 96 h. These cells were dissociated and subjected to single-cell RNA-seq by Annoroad Gene Technology Co., Ltd. (Beijing, China), and single-cell suspensions (approximately 1000 viable cells/mL, CellDrop FL (DeNovix Inc. Wilmington, DE, USA)) were loaded onto a Chromium chip using 3′ GEM Kit v3.1 (10× Genomics, Pleasanton, CA, USA) per protocol. cDNA was synthesized (Veriti PCR Thermal Cyclers: 53 °C for 45 min, 85 °C for 5 min, and held at 4 °C) and sequenced on an Illumina platform. Reads were processed by the Cell Ranger (v7.1.0, 10× Genomics) with the Sus scrofa 11.1 genome, and doublets were removed using Scrublet/DoubletFinder. A seurat object (v4.3.0, R v4.0.3) was created with the following filters [32]: genes expressed in <3 cells, cells with 200 to 10,000 genes, and cells with ≤20% mitochondrial UMIs.

After filtering (14,822 cells retained; median UMI: 24,142; median gene: 2595), counts were normalized (scale factor 10,000) and log-transformed, and the top 2000 HVGs were identified for integration. Single-cell RNA-seq data were deposited in the NCBI GEO database (accession number GSE315039). PCA was carried out using default parameters, where significant PCs were selected via the elbow method. Cell clustering was carried out using the Louvain algorithm [33] and visualized with uniform manifold approximation and projection (UMAP) [34]. Differential/marker genes were conducted using ‘findMarkers’ (Wilcoxon’s test: min.pct = 0.1, logfc.threshold = 0.25). Clusters were annotated using SciBet (v1.0) and pseudotime analysis was performed on cell clusters using the Monocle3 R package (https://cole-trapnell-lab.github.io/monocle3, accessed on 9 April 2025) [35].

### 2.15. Statistical Analysis

Data are presented as the mean ± standard deviation (X ± SD). Statistical analyses were performed via GraphPad Prism software (version 9.0, GraphPad Software, San Diego, CA, USA). Two-group comparisons were carried out using Student’s t-test, and multiple group comparisons were carried out using one-way ANOVA. *p* < 0.05 was considered significant.

## 3. Results

### 3.1. Derivation and Characterization of Porcine EPSCs

#### 3.1.1. Derivation of Porcine EPSCs

**Figure 1:**
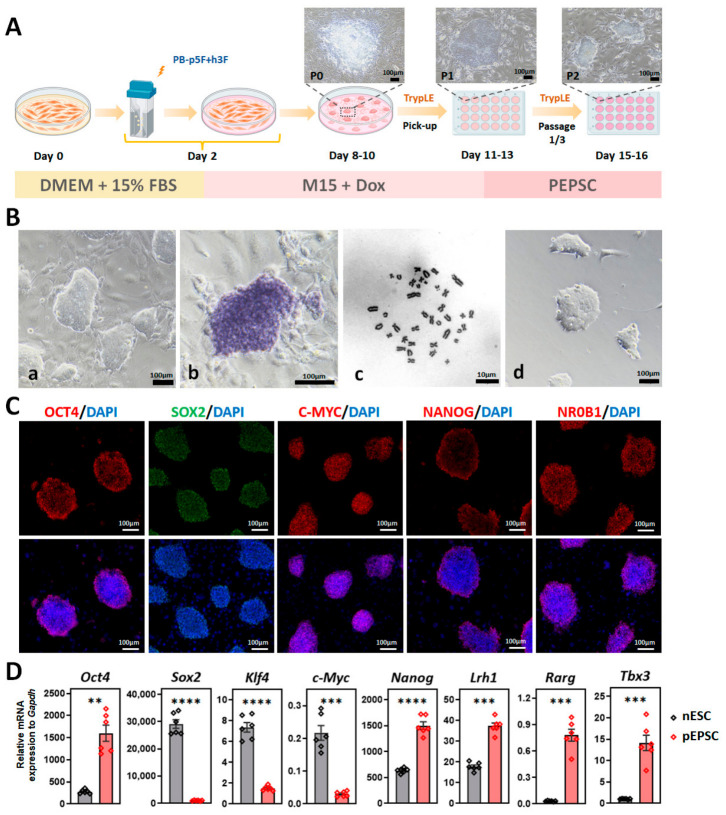
Establishment and characterization of porcine EPSCs (pEPSCs). (**A**) Schematic diagram of pEPSCs derivation from PEFs via reprogramming and culture system transition. PB-p5F+h3F, eight exogenous reprogramming factors including PB-Tre-pOSKM (porcine *Oct4*, *Sox2*, *Klf4*, and *c-Myc*), PB-Tre-pNhL (porcine *Nanog* and human *LIN28*), PB-Tre-hRL (human *RARG* and *LRH1*); M15, M15 medium supplemented with 15% FBS and 10 ng/mL hLIF; Dox, doxycycline; PEPSC, pEPSCs conversion medium. Scale bar, 100 μm. (**B**) Morphology, alkaline phosphatase (AP) staining, and karyotype analysis of pEPSC-30#. (**a**) Colony morphology on STO feeder cells at passages 29; (**b**) AP staining; (**c**) karyotype analysis; (**d**) feeder-free culture at passages 18. (**C**) Immunofluorescence staining of OCT4, SOX2, C-MYC, NANOG, and NR0B1 in pEPSCs. Scale bars, 100 μm. (**D**) RT-qPCR analysis of pluripotency gene expression in pEPSCs (*n* = 3) and porcine nESCs (*n* = 2). Data are presented as the mean ± SD. Statistical significance: ** *p* < 0.01, *** *p* < 0.001, **** *p* < 0.0001.

To derive porcine EPSCs, we transfected PEFs with eight exogenous reprogramming factors (p5F+h3F: porcine *Oct4*, *Sox2*, *Klf4*, *c-Myc*, and *Nanog*, along with human *LIN28*, *RARG*, and *LRH1*) via the piggyBac system. After ~1 week of induction in doxycycline (Dox)-supplemented M15 medium, 0.054% of transfected PEFs (697 out of 1.3 × 10^6^) formed primary colonies (Figure 1A and Figure S1A), which were picked, dissociated with TrypLE, and passaged onto STO feeders in M15 medium. Most cells retained the exogenous reprogramming factors and expressed endogenous core pluripotency genes (*Oct4*, *Sox2*, and *Nanog*; Figure S1B,C). They were transferred to PEPSC medium (supplemented with CHIR99021, WH-4-023, XAV939, Activin A, and hLIF) and >2/3 colonies were converted into EPSCs, with their morphology shifting from loose to dense clusters within two passages (P0 to P2; Figure 1A).

Seven stable pEPSC lines were established from 20 initial colonies (35%), maintaining self-renewal for over 50 passages (split ratios: 1:3 to 1:30–40). Core pluripotency genes (Oct4 and Dppa5) were more highly expressed in PEPSC medium than M15 medium (Figure S1D). Cells were alkaline phosphatase (AP)-positive, maintained a normal 38XX karyotype, and could be cultured under feeder-free conditions (Figure 1B and Figure S1E). Immunofluorescence confirmed that these cells expressed the core pluripotency markers OCT4, SOX2, C-MYC, NANOG, and NR0B1 (Figure 1C). Compared to porcine naïve ESCs, pEPSCs exhibited significantly higher expression of *Oct4*, *Nanog*, *Lrh1*, *Rarg*, and *Tbx3*, which are markers associated with naïve pluripotency (Figure 1D) and consistent with a more naïve state under the pEPSC medium condition.

#### 3.1.2. Transcriptomic Profiling Reveals EPSC-Specific Signatures

**Figure 2:**
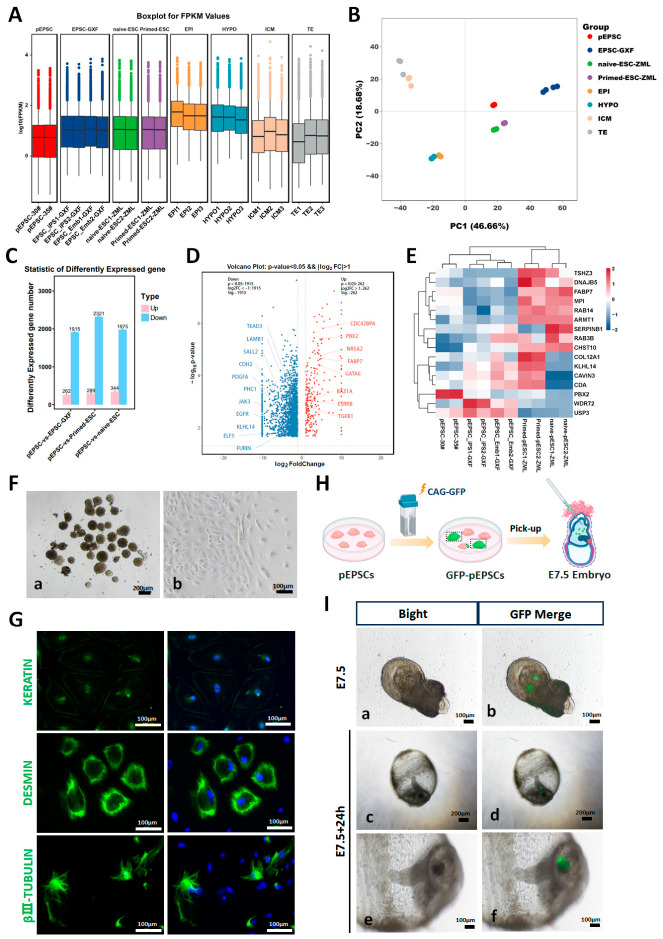
Transcriptomic profiling, differentiation capacity, and developmental potential of pEPSCs. (**A**) Gene expression distribution in pEPSCs (pEPSC-30# P18 and pEPSC-35# P15) and publicly available datasets: porcine EPSC-GXF (piPSC_EPSC1, piPSC_EPSC2, pEPSC1, and pEPSC2 in GSE129760), naïve ESC (naïve-ESC-ZML; nESC1 and nESC2 in GSE183270), primed ESC (primed-ESC-ZML; pESC1 and pESC2 in GSE183270), and epiblast (EPI; LB-EPI in GSE112380), hypoblast (HYPO; LB-HYPO in GSE112380), inner cell mass (ICM; ICM-1, ICM-2 and ICM-3 in GSE139512) and trophectoderm (TE; TE-1, TE-2 and TE-3 in GSE139512). (**B**) Global transcriptome profiling of different cell types and embryonic lineages. (**C**) Differential gene expression analysis between our pEPSCs and porcine EPSC-GXF, naïve ESCs, and primed ESCs. (**D**) Main differentially expressed genes between our pEPSCs and porcine EPSC-GXF. (**E**) Heatmap of differentially expressed genes between human EPSCs and primed PSCs, comparing across our pEPSCs, porcine EPSC-GXF, naïve ESCs, and primed ESCs. (**F**) Morphology of embryoid body (EB)-like spheroids and differentiated cells derived from the EB-like spheroids. (**a**), EB-like spheroids derived from pEPSC-30# cells at passage 18; (**b**), EB-like spheroids spreading on gelatin-coated dishes showing the three-germ-layer differentiation. (**G**) Immunofluorescence staining of the differentiation markers KERATIN, DESMIN, and βIII-TUBULIN in pEPSCs-derived differentiated cells. Scale bar, 100 µm. (**H**) Schematic of xenogeneic chimeric embryo generation: CAG-GFP-transfected pEPSCs were injected into E7.5 mouse embryos (~15 cells per embryo). (**I**) Microscopic imaging of GFP-pEPSCs contribution in mouse late embryos. (**a**,**b**), E7.5 mouse embryos after GFP-pEPSCs injection; (**c**–**f**), in vitro cultured chimeric embryos 24 h post-injection of GFP-pEPSCs. Scale bar, 200 µm (**a**–**c**,**e**), and 100 µm (**d**,**f**) respectively.

To investigate the transcriptional profile of pEPSCs, we performed RNA sequencing on two pEPSC lines (pEPSC-30# at P18, pEPSC-35# at P15), which showed high inter-line consistency among 17,009 detected genes (Figure 2A). Global expression profiling indicated that pEPSCs clustered closely with the previously reported porcine EPSC-GXF [4], and were distinct from porcine naïve/primed ESCs25 and blastocyst lineages (EPI, HYPO, ICM, and TE) [30,31] (Figure 2B). The correlation heatmap confirmed that pEPSCs correlated more strongly with porcine ESCs (EPSC-GXF and naïve/primed ESCs) than with blastocyst lineages (Figure S2A).

Differential expression analysis between pEPSCs and porcine EPSC-GXF identified 262 genes that were upregulated (including pluripotency-associated genes *Cdc42bpa*, *Pbx2*, and *Gata6*) and 1915 that were downregulated (including the differentiation markers *Tead3*, *Sall2*, and *Cdh2*) out of 5826 that were analyzed (Figure 2C,D). Compared to primed ESCs, 289 genes were upregulated (e.g., pluripotency genes *Efr3a*, *Pbx2*, and *Hspa8*) and 2321 were downregulated (e.g., differentiation genes *Wnt1*, *Lck*, and *Krt18*) among 5657 (Figure 2C and Figure S2B). In comparison with naïve ESCs, 344 genes were upregulated (e.g., pluripotency genes *Nr5a2*, *Pbx2*, and *Gsc*) and 1975 were downregulated (e.g., differentiation genes *Igfbp7*, *Egf*, and *Tgfbr3*) out of 5588 (Figure 2C and Figure S2C). We performed KEGG pathway enrichment analysis to further investigate how these differentially expressed genes might rewire signaling. Our results revealed that: compared to porcine EPSC-FXF, upregulated genes were enriched in MAPK signaling, while downregulated genes were associated with TGF-β signaling (Figure S2D); when compared to naïve/primed ESCs, upregulated genes were enriched in Notch, TGF-β, and mTOR signaling pathways (Figure S2E,F).

Moreover, compared to the established lineages of natural blastocysts (ICM, EPI, TE, and HYPO) [30,31], pEPSCs showed gene expression profiles—especially for core pluripotency genes and TE-associated genes—that clustered more closely with the ICM than with the TE lineage (Figure S2G–I). pEPSCs exhibited human EPSC-like expression patterns [36] (Figure 2E) and high expression of canonical pluripotency markers such as *Nr5a2*, *Esrrb*, and *Gata6* (Figure S2G). TE/HYPO-related genes were significantly lower in pEPSCs than in embryonic lineages (Figure S2H,I), consistent with the porcine ICM or totipotent-like transcriptional profile [31]. Collectively, these data demonstrate that we successfully derived pEPSC lines exhibiting pluripotency and an EPSC-like transcriptional profile.

#### 3.1.3. Three-Germ-Layer Differentiation from Porcine EPSCs

To assess in vitro differentiation capacity, pEPSCs were induced to form embryoid bodies (EBs) by a 7-day hanging-drop culture and a 3-day adherent culture (no hLIF and small molecule inhibitors) (Figure 2F). RT-qPCR analysis revealed significant upregulation of the germ layer-specific markers in the EB-derived spheroids compared to undifferentiated pEPSCs: endodermal (*Ncstn*, *Albumin*), mesodermal (*Myh11*, *Desmin*), and ectodermal (*Nefl*, *Nestin*) (Figure S2J). Immunofluorescence staining further confirmed the expression of germ layer-specific protein markers—KERATIN (endoderm), DESMIN (mesoderm), and βШ-TUBULIN (ectoderm)—in differentiated cells (Figure 2G). Therefore, pEPSCs can differentiate into all three germ layers in vitro.

#### 3.1.4. Cross-Species Chimeric Competence of Porcine EPSCs

We further assessed the developmental potential of pEPSCs through a cross-species chimeric embryo assay (Figure 2H). To better monitor the chimeric contribution of pEPSCs in mouse embryos, green fluorescent protein (GFP)-labeled pEPSCs (GFP-pEPSCs) were generated by transfecting the CAG-GFP plasmid into pEPSC-30# at passage 16. Approximately 15 GFP-pEPSCs were microinjected into the distal region of E7.5 mouse embryos at the late gastrula stage, and 75% (21 in 28, from five pregnant mice) of these injected mouse embryos were subsequently cultured in vitro for 24 h. Microscopic examination revealed that 56.25% (9 in 16 survival embryos) of developing host embryos had successful GFP-pEPSC integration into the neural ectoderm (Figure 2I). During the subsequent 48 h, the chimeric embryos progressively underwent developmental arrest or death, with some exhibiting primitive heart tube beating. Immunofluorescence area quantification indicated that the contribution rate of pEPSCs to the chimeric mouse embryos ranged from approximately 2% to 5%. These data demonstrate that pEPSCs can contribute to cross-species chimeras during development of the mouse neurula stage.

### 3.2. Transient Coexistence of Trilineage in LCDM

#### 3.2.1. Differential Characteristics of Porcine EPSCs in LCDM

**Figure 3:**
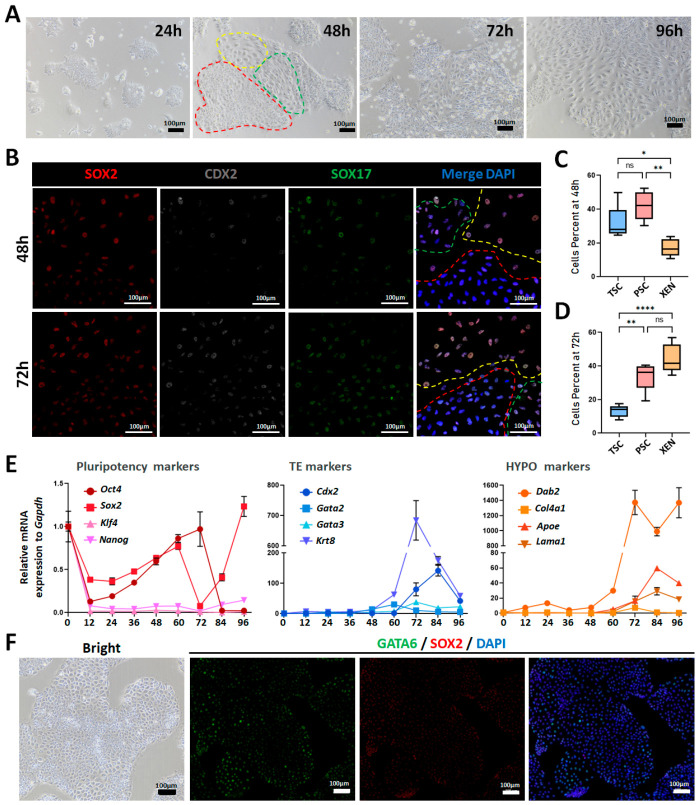
Characterization of pEPSCs differentiated in LCDM system. (**A**) Morphology of three lineage stem cells (PSCs, TSCs, and XEN cells) differentiated from pEPSCs in LCDM medium at 24, 48, 72, and 96 h. Red dashed circle, PSCs; green, TSCs; yellow, XEN cells. Scale bar, 100 µm. (**B**) Immunofluorescence staining of the lineage markers (SOX2, CDX2, and SOX17) in differentiated cells in LCDM at 48 and 72 h. Scale bar, 100 µm. (**C**,**D**) Quantitative analysis of PSC (SOX2^low^/CDX2^low^/SOX17^low^), XEN (SOX2^high^/CDX2^high^/SOX17^high^), and TSC (SOX2^low^/CDX2^high^/SOX17^low^) cell percentages at 48 and 72 h. Data are presented as the mean ± SD. Statistical significance: * *p* < 0.05, ** *p* < 0.01, **** *p* < 0.0001, ns, not significant. (**E**) RT-qPCR analysis of the core pluripotency (*Oct4*, *Sox2*, *Klf4*, and *Nanog*), TE lineage (*Cdx2*, *Gata2*, *Gata3*, and *Krt8*), and HYPO lineage (*Dab2*, *Col4a1*, *Apoe*, and *Lama1*) gene expression during 0–96 h differentiation in LCDM medium. (**F**) Morphology and immunofluorescence staining of differentiated cells after two passages in LCDM. Scale bar, 100 µm.

In our previous study, we observed that when ICMs from E6 porcine blastocysts were cultured in LCDM medium, two distinct clone types with EPI-like and HYPO-like morphologies emerged simultaneously [25]. We cultured the feeder-free pEPSCs in the LCDM system, and found that they undergo three morphologically distinct cell types at 48–72 h: (i) small, dense PSC-like cells (abbreviated as PSCs); (ii) round, loose XEN-like cells (abbreviated as XEN cells), and (iii) thin, dispersed TSC-like cells (abbreviated as TSCs; Figure 3A). Immunofluorescence confirmed lineage identity: PSCs (SOX2^low^/CDX2^low^/SOX17^low^), XEN cells (SOX2^high^/CDX2^high^/SOX17^high^), and TSCs (SOX2^low^/CDX2high/SOX17^low^) (Figure 3B). Quantitative analysis revealed that at 48 h, PSCs and TSCs were predominant with comparable abundances, while there were significantly fewer XEN cells (Figure 3C). At 72 h, XEN cells and PSCs constituted the majority, whereas TSCs decreased markedly (Figure 3D).

RT-qPCR analysis monitored dynamic changes in the expression of core pluripotency and lineage-specific genes over 0–96 h in LCDM culture. *Oct4*, *Sox2*, *Klf4*, and *Nanog* (core pluripotency) decreased sharply at 12 h; partial *Oct4* and *Sox2* recovered at 36 h and declined at 60–72 h; and Sox2 was upregulated at 84 h. TE genes (*Cdx2*, *Gata2*, *Gata3*, and *Krt8*) increased at 36 h, peaked at 60–84 h, and fell to the lowest point at 96 h. HYPO genes (*Dab2*, *Col4a1*, *Apoe*, and *Lama1*) were upregulated at 48 h, peaked at 72–84 h, and elevated the most at 96 h, except *Col4a1* (Figure 3E). Immunofluorescence analysis of pEPSCs undergoing differentiation in LCDM at the specified time points showed a progressive downregulation of OCT4 expression from initially high to low levels. The emergence of GATA3-expressing cells was observed between 36 and 48 h, reaching a peak at 60 h, followed by a gradual decrease. The HYPO marker GATA6 expression commenced at 60 h and progressively increased, eventually becoming the dominant population (Figure S3A,B). These findings were largely consistent with the RT-qPCR results, confirming the dynamic alterations in the relative proportions of PSC, TSC, and XEN cell lineages.

After passaging in LCDM, XEN cells gradually predominated in the subsequent culture processes, and the heterogeneous cells adopted uniform XEN-like morphology, expressing GATA6 with weak SOX2 and CDX2 (Figure 3F and Figure S3C).

#### 3.2.2. Differential Trajectory of Porcine EPSCs in LCDM

**Figure 4:**
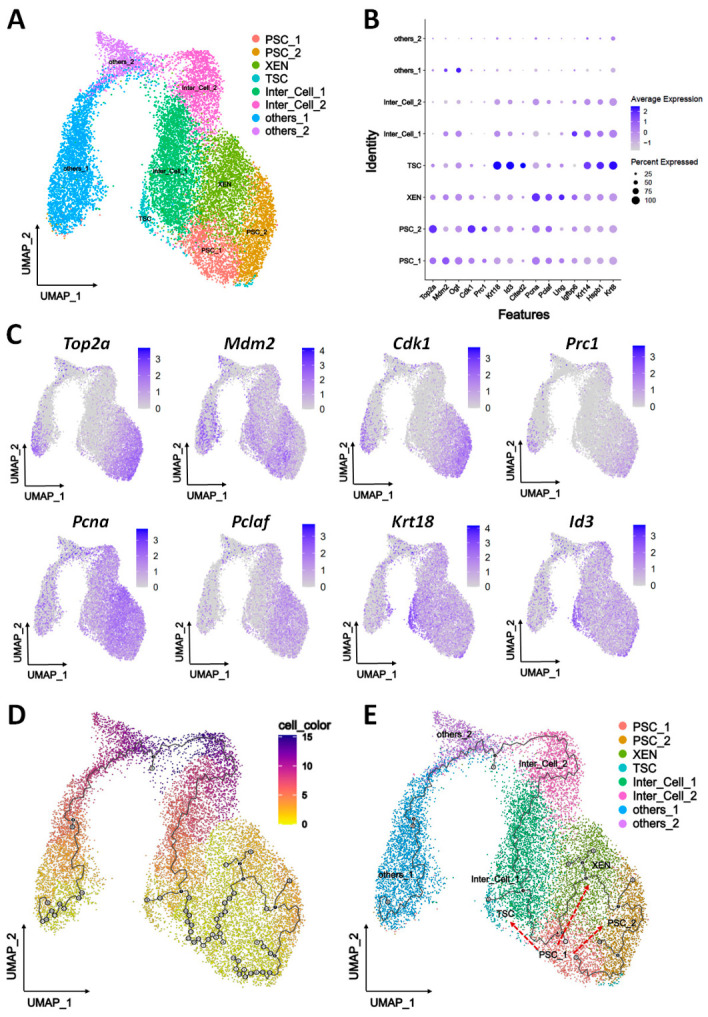
Single-cell transcriptomic atlas and developmental trajectories of pEPSCs differentiated in LCDM. (**A**) UMAP visualization of eight major cell types: PSC_1 and PSC_2 (PSCs), XEN (XEN cells), TSC (TSCs), Inter_Cell_1 (PSC-TSC intermediate), Inter_Cell_2 (TSC-XEN intermediate). (**B**) Dot plot showing the expression level (color scale) and percentage of expressing cells (dot size) for the marker genes across cell types. (**C**) UMAP plots colored by expression levels of *Top2a*, *Cdk1*, *Mdm2*, *Prc1*, *Pcna*, *Pclaf*, *Krt18* and *Id3* genes. Purple to gray: high to low expression. (**D**) Cell trajectory analysis of pEPSCs differentiated in LCDM during 0–96 h. Color gradient indicates pseudotime differentiation from low (bright) to high (dark). (**E**) Diffusion pseudotime analysis of eight cell clusters, illustrating the differentiation process in LCDM medium. Red dashed arrows indicate the direction of cell differentiation.

To achieve higher-resolution and unbiased characterization of the cell types generated under the LCDM condition, we performed single-cell RNA-sequencing (scRNA-seq) of pEPSC cultures. Following quality control, the 10× Genomics scRNA-seq dataset of 14,822 cells harvested at 96 h yielded 14,821 high-quality cells with a median of 2595 genes detected per cell (Table S4). Network clustering analysis identified eight distinct cell clusters (Figure S4A,B), of which six were annotated via the lineage markers and porcine blastocyst datasets [30,31]: PSCs (PSC_1 and PSC_2), XEN cells (XEN), TSCs (TSC), and intermediate (Inter_Cell_1: PSC-TSC; Inter_Cell_2: TSC-XEN) cells (Figure 4A and Table S5).

Clusters were characterized by marker expression: PSC_1 with the 2-cell-like marker *Top2a* [37], the early ICM marker *Mdm2* [38], the ESC marker *Ogt* [39] and the pluripotency markers *Esrrb* and *Gata6*; PSC_2 with *Top2a* and the ESC markers *Cdk1* [40,41] and *Prc1* [42]; XEN with the proliferative markers *Pcna* [43], its co-factor *Pclaf*, the yolk sac marker *Ung* [44], and HYPO lineage marker *Gata6* and *Sall4* [25]; and TSC with the TE markers *Krt18*, *Krt8*, and *Id3* [45,46,47] (Figure 4B,C and Figure S4C–E). Furthermore, Monocle 3 pseudotime analysis showed that PSC_1 cells were the original, and they differentiated toward PSC_2, XEN, and TSC, with partial progression to intermediate states, Inter_Cell_1 and Inter_Cell_2 (Figure 4D,E).

At 96 h, the population retained heterogeneity with TSC-like cells <2% and mostly intermediate states (Table S4), confirming the multidirectional differentiation and lineage coexistence of pEPSCs in the LCDM system. These results indicate that pEPSCs in LCDM undergo parallel differentiation, with a transient coexistence of trilineage (PSCs, TSCs, and XEN cells) between 36 and 84 h, simulating early porcine embryonic development in vitro.

### 3.3. XEN Cells and TSCs Differentiated from Porcine EPSCs in LCDM

#### 3.3.1. Hypoblast Lineage Differentiation of Porcine EPSCs in Prolonged LCDM Culture

**Figure 5:**
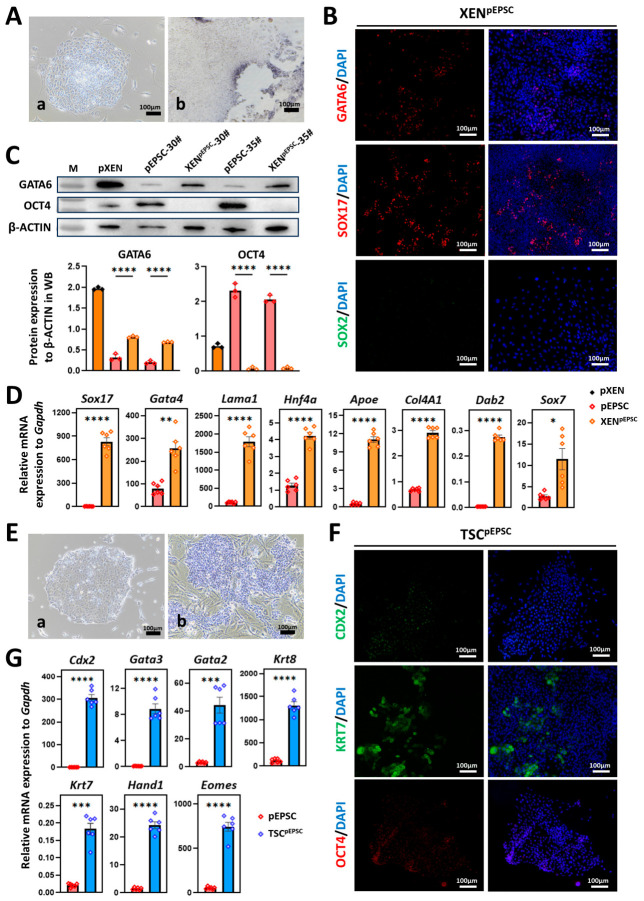
Differentiation of HYPO- and TE-lineage stem cells from pEPSCs (XEN^pEPSC^ and TSC^pEPSC^ lines). (**A**) Morphology and AP staining of XEN^pEPSC^. (**a**) XEN^pEPSC^ colony on gelatin at passages 5; (**b**) AP staining of XEN^pEPSC^ on gelatin at passages 7. Scale bars, 100 μm. (**B**) Immunofluorescence staining of the HYPO lineage markers (GATA6 and SOX17) and the core pluripotency marker SOX2 in XEN^pEPSC^ colonies. Scale bar, 100 µm. (**C**) Western blot and quantitative analysis of GATA6 and OCT4 in pXEN cells, pEPSCs and XEN^pEPSC^ cells. β-ACTIN was a loading control. (**D**) RT-qPCR analysis of the HYPO lineage markers (*Sox17*, *Gata4*, *Lama1*, *Hnf4a*, *Apoe*, *Col4a1*, *Dab2*, and *Sox7*) in pEPSCs and XEN^pEPSC^ cells. (**E**) Morphology and AP staining of TSC^pEPSC^ lines. (**a**) TSC^pEPSC^ colony on Geltrex at passages 6; (**b**) AP staining of TSC^pEPSC^ on STO feeder cells at passages 6. Scale bars, 100 μm. (**F**) Immunofluorescence staining of the TE lineage marker (CDX2 and KRT7) and the core pluripotency marker OCT4 in TSC^pEPSC^ colonies. Scale bar, 100 µm. (**G**) RT-qPCR analysis of the TE lineage markers (*Cdx2*, *Gata3*, *Gata2*, *Krt8*, *Krt7*, *Hand1*, and *Eomes*) in pEPSCs and TSC^pEPSC^. Data are presented as the mean ± SD. Statistical significance: * *p* < 0.05, ** *p* < 0.01, *** *p* < 0.001, **** *p* < 0.0001.

As discussed above, pEPSCs were eventually differentiated into XEN^pEPSC^ cells after 8 days (two passages) in prolonged LCDM culture, and these XEN^pEPSC^ cells were stably cultured in gelatin-coated dishes for over 10 passages (Figure S5A). Colonies were AP-positive with a large and flat morphology and normal karyotype (Figure 5A and Figure S5A,B). Immunofluorescence and Western blot analyses confirmed the upregulation of the HYPO lineage markers (GATA6 and SOX17), and downregulation of the core pluripotency markers (OCT4, SOX2, and NANOG), compared to undifferentiated pEPSCs (Figure 5B,C and Figure S5C,D). RT-qPCR revealed an elevated expression of HYPO lineage genes (*Sox17*, *Gata4*, *Hnf4a*, *Lama1*, *Sox7*, *Col4a1*, *Dab2*, and *Apoe*) and lower expression of *Oct4*, *Sox2*, *Nanog*, and *Klf4* (Figure 5D and Figure S5E). These results showed the XEN^pEPSC^ cells aligned with our previous report on porcine embryonic XEN cells [25].

#### 3.3.2. More Efficient Trophoblast Lineage Commitment Through LCDM Culture

As discussed above, the TSCs could be partially obtained during pEPSC differentiation in the LCDM system for 0–96 h. To further determine how long a culture in LCDM is more conducive to the stable and efficient differentiation of EPSCs into TSCs, we attempted to insert a brief LCDM induction (24, 48, and 72 h) before the human TSC-adapted system (3% FBS, TGF-β inhibitor SB431542 and A83-01, and hEGF) [48], and found that undergoing 48 h of LCDM culture before differentiating into TSCs enables earlier and more stable TSC^pEPSC^ cells (Figure S6A). Then, pEPSCs were differentiated into TSC^pEPSC^ after 12 days (three passages) in the TSC system with 3% FBS, while 0.3% FBS caused cell detachment and excessive differentiation (Figure S6B). This finding suggests that a slightly higher serum concentration both promoted the adherent growth of TSCs and helped preserve their three-dimensional clonal morphology. Moreover, unlike the inefficient long-term passaging observed with STO feeder layers, Geltrex-coated dishes provided a more effective substrate for TSC.

TSC^pEPSC^ were cultured in Geltrex-coated dishes for over 8 passages. Colonies were AP-positive with TE-like morphology and normal karyotype (Figure 5E and Figure S6C), and immunofluorescence confirmed expression of the TE markers (CDX2 and KRT7) and residual core pluripotency markers (OCT4 and SOX2), but not the HYPO markers (SOX17 and GATA6) (Figure 5F and Figure S6D). Relative to undifferentiated pEPSCs, TSC^pEPSC^ had higher CDX2 and KRT7, and lower OCT4, GATA6, and SOX17 (Figure S6E,F), and RT-qPCR revealed upregulated TE markers (*Cdx2*, *Gata3*, *Gata2*, *Krt8*, *Krt7*, *Hand1*, and *Eomes*) and downregulated *Oct4* and *Nanog* (Figure 5G and Figure S5E), consistent with porcine TE characteristics [49].

### 3.4. Primed PSCs Differentiated from Porcine EPSCs

**Figure 6:**
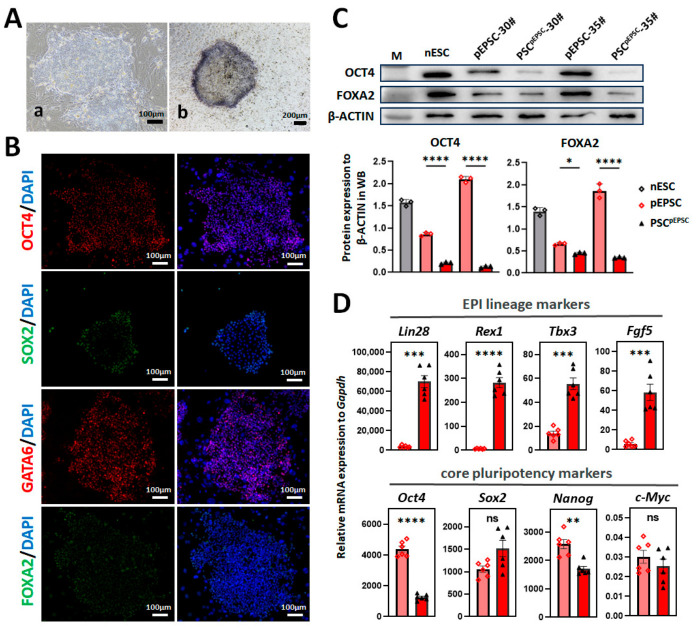
Differentiation of EPI-lineage stem cells from pEPSCs (primed PSC^pEPSC^). (**A**) Morphology and AP staining of primed PSC^pEPSC^ colonies. (**a**) Primed PSC^pEPSC^ colonies on STO feeder cells at passages 12; Scale bars, 100 μm. (**b**) AP staining of primed PSC^pEPSC^ at passages 12; Scale bars, 200 μm. (**B**) Immunofluorescence staining of the pluripotency markers (OCT4, SOX2, GATA6, FOXA2, and NR0B1) in primed PSC^pEPSC^ colonies. Scale bar, 100 µm. (**C**) Western blot and quantitative analysis of OCT4 and FOXA2 expression in naïve ESCs (nESCs), pEPSCs, and primed PSC^pEPSC^. β-ACTIN was a loading control. (**D**) RT-qPCR analysis of the EPI lineage markers (*Lin28*, *Rex1*, *Tbx3*, and *Fgf5*) and the core pluripotency genes (*Oct4*, *Sox2*, *Nanog*, and *c-Myc*) in pEPSCs and primed PSC^pEPSC^. Data are presented as the mean ± SD. Statistical significance: * *p* < 0.05, ** *p* < 0.01, *** *p* < 0.001, **** *p* < 0.0001.

Under porcine ESC conditions [28], pEPSCs differentiated into primed PSC^pEPSC^, transitioning from three-dimensional colonies to a flattened epithelial monolayer within 4 passages (16 days). Cells were AP-positive and exhibited a normal karyotype (Figure 6A and Figure S7A). Immunofluorescence and Western blot confirmed the expression of the pluripotency markers (OCT4, SOX2, NANOG, GATA6, FOXA2, and NR0B1) (Figure 6B,C and Figure S7B). Compared to undifferentiated pEPSCs, primed PSC^pEPSC^ showed lower OCT4, SOX2, NANOG, and NR0B1 and higher GATA6 (Figure 6C and Figure S7C). RT-qPCR indicated that primed PSC^pEPSC^ upregulated pluripotency gene Klf4 and the EPI lineage markers *Lin28*, *Rex1*, *Tbx3*, and *Fgf5*, and downregulated core pluripotency genes *Oct4 and Nanog* (Figure 6D). Primed PSC^pEPSC^ lines also expressed higher genes associated with LIF/STAT3 (*Lifra* and *Lifrb*), FGF (*Fgf* and *Fgfr2*), and ACTIVIN/NODAL pathways (Figure S7D). These results confirmed that pEPSCs could be differentiated into EPI lineage stem cells in a porcine ESC culture system [28,29].

### 3.5. PGC Induction Potential of Porcine EPSCs

**Figure 7:**
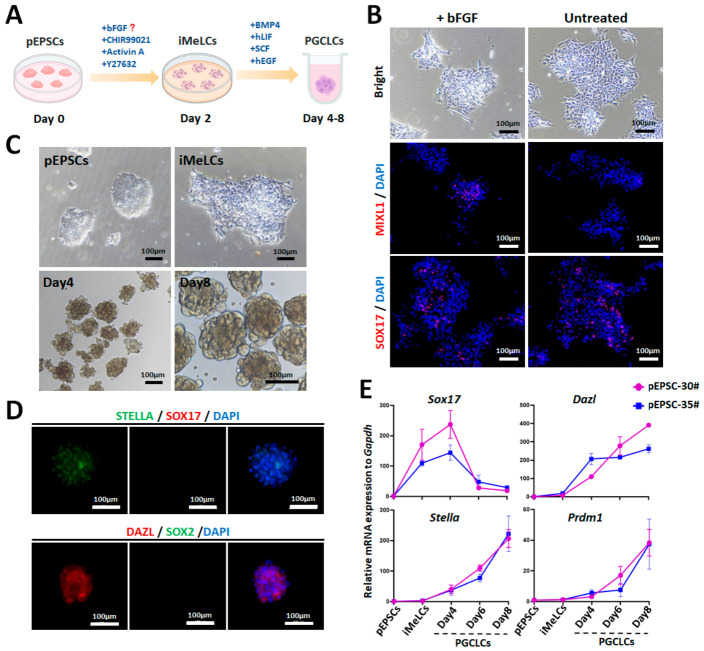
Derivation and characterization of PGCLCs differentiated from pEPSCs. (**A**) Schematic of PGCLCs generation through iMeLCs induction. The red question mark indicates that there is uncertainty regarding whether need bFGF supplement during iMeLCs induction. (**B**) Morphology and immunofluorescence staining of the mesodermal marker MIXL1 and the early PGCLCs marker SOX17 in iMeLCs with or without bFGF treatment. Scale bar, 100 µm. (**C**) Morphology of pEPSCs, iMeLCs, and PGCLCs at day 4 and day 8. Scale bar, 100 µm. (**D**) Immunofluorescence staining of the core pluripotency marker SOX2 and the PGC markers (SOX17, STELLA, and DAZL) in PGCLCs at day 8. Scale bar, 100 µm. (**E**) RT-qPCR analysis of the key PGC specification genes (*Sox17*, *Stella*, *Prdm1*, and *Dazl*) in pEPSCs, iMeLCs and PGCLCs (at day 4, 6, 8). Data are presented as the mean ± SD.

The PGC induction potential of EPSCs reflects their pluripotency and determines their application prospects in the research of reproductive development, infertility treatment, and transgenic animal production. Using a human PGCLC protocol [27] with bFGF for inducing incipient mesoderm-like cells (iMeLCs) identity and PGC competence [50], pEPSCs (pEPSC-30# at P19 and pEPSC- 35# at P16) were differentiated into iMeLCs for 2 days, which showed an epithelial-like monolayer with distinct cell boundaries (bFGF enhanced three-dimensional organization; Figure 7A,B). Immunofluorescence confirmed SOX17 (the endoderm marker) and higher MIXL1 (the early primitive streak marker) in bFGF-treated cells.

In U-shaped microwells with BMP4, SCF, hEGF, and hLIF, iMeLCs formed nest-like PGCLC colonies by days 4–8 (Figure 7C). Immunostaining confirmed the PGC markers (STELLA and DAZL) on day 8, but not the core pluripotency marker SOX2 or the early PGC marker SOX17 (Figure 7D). RT-qPCR revealed dynamic marker patterns: gradual upregulation of the early PGC markers (*Stella* and *Prdm1*), and sharp upregulation of the late PGC (oogonia/gonocyte) marker *Dazl*, with an initial upregulation and then decline in *Sox17* (Figure 7E). These results confirmed that our pEPSCs showed porcine PGC induction potential without any exogenous support [51,52]. Meanwhile, the expression pattern of *Sox17*, *Stella*, and *Dazl* was consistent with mice PGCLCs [53], and the repression of SOX2 and the expression of *Prdm1* were more similar to human PGC specification and development in vitro [54].

## 4. Discussion

EPSCs exhibiting certain totipotent features can be established through two main strategies: inhibiting first-lineage differentiation pathways during early cleavage stages, or modulating signaling pathways using chemical small molecules in ESC or iPSC culture systems. Gao et al. developed a universal system for porcine/human EPSC derivation [4], and we generated pEPSCs from PEFs using a modified version of this protocol. In contrast to the previous protocol, which used EPSC medium with multiple small molecules for a reprogramming experiment [4], we cultured the primary colonies in DOX-supplemented M15 medium initially and made a stable transition into the EPSC system within 1–2 passages. These pEPSCs exhibited a characteristic colony morphology, strong core pluripotency marker expression, and three-germ-layer differentiation capacity, which were consistent with the characteristics of EPSC-GXF [4]. Unlike the previous study that only explored the differentiation potential of EPSCs toward the TE lineage [4,55], our study extended these findings by successfully demonstrating the differentiation toward EPI and XEN lineages. Importantly, we achieved PGCLC differentiation without any exogenous support—a process that previously required exogenous SOX17 assistance [4]. Meanwhile, transcriptomic profiling aligned our pEPSCs with previously reported porcine EPSC-GXF [4], while exhibiting unique traits. Like EPSC-GXF, our pEPSCs upregulated Notch and mTOR pathway components vs. naïve/primed ESCs—pathways critical for pluripotency [56,57]. Notch maintains progenitor stemness via effectors (e.g., HES1) and modulates cell fate [58,59], and mTOR coordinates metabolism to regulate pluripotent states [60]. Our pEPSCs also displayed elevated MAPK signaling, a pathway with context-dependent roles in self-renewal and differentiation [61]. We propose that a balanced crosstalk between these pathways underlies their high-density colonies, stable long-term passaging, and efficient multilineage differentiation.

Historically, porcine ESCs’ instability limited their utility in chimeras [62], and EPSC-like porcine iPSCs showed low chimerism (≤0.04%) in mouse/porcine blastocysts [63]. By contrast, our pEPSCs integrated robustly into mouse embryos, likely due to a high expression of extended pluripotency markers (e.g., Lrh1 and Rarg). Their contribution to neurula-stage embryos suggests responsiveness to neuroectoderm-inducing signals during late gastrulation [64], supporting use in early developmental studies. We also induced pEPSC differentiation into PGCLCs—a foundation for porcine in vitro gametogenesis. While mouse PGC specification requires BMP4 [65] and human PGC induction involves BMP4 and bFGF [66], bFGF alone was sufficient for porcine PGCLC induction. Notably, SOX17 (essential for human PGC fate) was undetectable in mature porcine PGCLCs; its impact on gametogenic competence requires further study. Regardless, strong xenogeneic chimerism capacity and efficient PGCLC differentiation make these pEPSCs ideal donors for human–pig chimera models, which advance research on germline development and organogenesis.

The LCDM system supports EPSC derivation across species [3,63,67]. Here, pEPSCs in LCDM showed transient trilineage (EPI/TE/HYPO) coexistence, with XEN-like cells emerging later than TE derivatives—recapitulating delayed HYPO specification in porcine embryos. Although medium switching could induce stress-related destabilization, the co-expression of the lineage markers at 36–84 h suggests that LCDM mirrors in vivo porcine lineage segregation to a certain extent. Transient upregulation of the Hippo pathway and NANOG-GATA6-FGF/ERK axis (the key regulators of mouse early fate decisions) implies evolutionary conservation [68,69]. CHIR99021-induced WNT activation and DIM-mediated MAPK inhibition likely synergized to promote TE/HYPO commitment, while LIF sustained PSC-like subpopulations. Cell fate transitions involve intermediate states, such as mesendodermal precursors during gastrulation [70,71,72]. Two intermediate clusters were also exhibited in LCDM at 96 h. Pseudotime analysis indicated these as plasticity ‘nodes’ with bidirectional differentiation potential during TE/HYPO specification. While LCDM transiently co-maintains EPI and HYPO lineages [25], extended culture favored XEN-like stabilization—likely due to MAPK inhibition (ERK downregulation) compromising TSC maintenance.

Lineage-specific differentiation of pluripotent stem cells is governed by distinct signaling networks [73,74,75]. The LIF/STAT3 pathway, essential for mouse ESC self-renewal but dispensable in human ESCs [76] and primed porcine ESCs [28,29], supported primed transition via feeder-derived LIF (no exogenous supplementation). Consistent with human ESC biology [77,78], FGF and ACTIVIN/NODAL signaling were critical for EPI lineage specification. For TSC induction, we adapted a human TSC-like protocol (WNT activation, EGF, and TGF-β inhibition)—diverging from mouse TSC requirements [79] and previous porcine TSC methods [49,80]. Our study indicated that for porcine early embryonic stem cells, the LCDM system (WNT activation and MAPK/AKT/mTOR inhibition) is more suitable for the long-term stable culture of XEN cells [25]. These results highlight the central role of WNT signaling in porcine pluripotency: low activity maintains pEPSCs, while high activity drives TE/HYPO commitment [81].

Large-animal blastoid research remains limited: only bovine blastoids (from EPSCs and TSCs) and porcine blastoids (from parthenogenetic blastocyst-derived ESCs) have been reported [18,19]. Developmentally competent blastoid models require a starting cell with trilineage differentiation potential (mimicking blastocyst cellular diversity and organization) and the ability to progress through gastrulation-like stages [82]. Our pEPSCs differentiate into all three blastocyst lineages and show a transient trilineage coexistence state in the LCDM. These findings provide both a cellular resource and mechanistic insights (e.g., key signaling pathways) for future porcine blastoid development and application in developmental studies.

## Data Availability

The data underlying this article are available in the article and in its online Supplementary Materials.

## Supplementary Materials

The following supporting information can be downloaded at: https://www.mdpi.com/article/10.3390/cells15100954/s1, Figure S1: Characterization of colonies cultured in M15 medium and PEPSC medium; Figure S2: RNA-seq analysis of pEPSCs and gene expression in EB-like from pEPSCs; Figure S3: Immunofluorescence characterization of pEPSCs differentiated in LCDM medium; Figure S4: Single-cell transcriptomic analysis of pEPSCs differentiated in LCDM medium; Figure S5: Derivation and characterization of XEN^pEPSC^ cells; Figure S6: Derivation and characterization of TSC^pEPSC^; Figure S7: Derivation and characterization of primed PSC^pEPSC^; Table S1: List of antibodies used in immunofluorescence analysis; Table S2: List of antibodies used in western blot analysis; Table S3: Primer information for RT-qPCR analysis; Table S4: CellRanger analysis statistics table of pEPSCs differentiated for 96 h in LCDM medium (namely pEPSC-LT); Table S5: Full names, abbreviations and proportions of different cell types of eight annotated clusters.
