# Supplementary material for: The Trilineage Coexistence Observed During the Differentiation of Porcine EPSCs
Source: Cells. 2026 May 21;15(10):954. doi: 10.3390/cells15100954 (PMC13204623; doi:10.3390/cells15100954)
Supplement: Supplementary file 1 [file cells-15-00954-s001.zip › Supplemental Tables.pdf]

Table S1. List of antibodies used in immunofluorescence analysis.

Antibody name	Brand	Cat No.	Dilution ratio	Proposed lineage
OCT4	Abcam	Ab18976	1:200	EPSCs [4,55], primed PSCs [28,29]
SOX2	CST	4900	1:400	EPSCs [4,55], primed PSCs [25]
SOX2	Abcam	Ab239218	1:50	
C-MYC	CST	D3N8F	1:200	EPSCs [55]
NANOG	CST	8822	1:500	EPSCs [4,55], primed PSCs [28,29]
NR0B1	Absin	Abs120562a	1:300	PSCs [29]
KERATIN	Millipore	MAB1677	1:400	endoderm [25,27,29]
DESMIN	Abcam	Ab227651	1:400	mesoderm [25,28,29]
βIII- TUBULIN	Abcam	Ab68193	1:100	ectoderm [25,27]
GATA6	CST	5851T	1:1600	EPSCs [31], XEN cells [19,25,55]
GATA3	Santa	SC-268	1:300	TSCs [4,19,49,55]
FOXA2	Abcam	Ab60721	1:200	EPSCs [4], primed PSCs [29]
CDX2	BioGenex	MU392A	1:200	TSCs [4,49,55]
KRT7	Santa	sc70936	1:400	TSCs [4,49,55]
SOX17	CST	81778	1:3200	XEN cells [25,81], early PGC [4,52]
MIXL1	proteintech	22772-1-AP	1:300	early primitive streak [52]
STELLA	Santa	sc376862	1:200	early PGC [51]
DAZL	Huabio	ET1704-75	1:200	late PGC [4,51]
Donkey anti-goat IgG H&L 568	Thermo	A11057	1:1000	
Donkey anti-mouse IgG Alexa Fluor 647	Thermo	A32787	1:2000	
Donkey anti-rabbit IgG H&L 488	Thermo	A21206	1:1000	
Goat anti-rabbit IgG H&L 546	ABclonal	AS007	1:1000	
Goat anti-mouse IgG Alexa Fluor 488	Thermo	A32723	1:1000	

Table S2. List of antibodies used in western blot analysis.

Antibody name	Brand	Cat No.	Dilution ratio	Proposed lineage
OCT4	Abcam	Ab18976	1:1000	EPSCs [4,55], primed

				PSCs [28,29]
FOXA2	Abcam	Ab60721	1:2000	EPSCs [4,55], primed PSCs [29]
GATA6	CST	5851T	1:1600	XEN cells [19,25,55]
β-ACTIN	Abclonal	A190	1:3000	
Goat Anti-Rabbit IgG H&L (HRP)	Abcam	Ab6721	1:10000	
HRP-conjugated Donkey anti-Mouse IgG (H+L)	Abclonal	AS003	1:10000	

Table S3. Primer information for RT-qPCR analysis.

Genes	Primer sequences（5'-3'）	Length (bp)	Proposed lineage
Oct4	F：AGCAGTGACTATTCGCAACG<br>R：CCAAAGCCCTGGTACAAAC	302	EPSCs [4,55], primed PSCs [28,29]
Sox2	F：GTGAGGGCCGGACAGTGAACTG<br>R：AAGCGTACCGGGTTTTTCTCCATAC	109	EPSCs [4,55], primed PSCs [28,29]
Klf4	F：GAACTGACCAGGCACTACCG<br>R：GACTTGTTGGGAACTTGACC	258	EPSCs [4,55]
c-Myc	F：TTACAACACCCGAGCGACAA<br>R：CCACTATCCGAAGGAAATCCAG	153	primed PSCs [28,29]
Nanog	F：CTGGAGGAGATCTTCATGATTCTAAG<br>R：AGTCCAGGCTTAAGTGTCTAGATAGAAG	56	EPSCs [4,55], primed PSCs [29]
Lin28	F：GTTCGGCTTCCTGTCCAT<br>R：CACAGCCTCACCCTCCTT	124	EPSCs [4,55], primed PSCs [28,29]
Rarg	F：AAGAGGTGAAGGAAGAAGGGTC<br>R：TGGAGTTCGTGGTGTATTTGC	138	EPSCs [4,55]
Lrh1	F：GAAAGGGATTGGTGGTGA<br>R：GCAATACAAATACCCTGATACA	203	EPSCs [4,55]
Rex1	F：GTCCTGAGAGTGGATGCACAAG<br>R：CTGTGAACGGAGAGATGCTTTCT	179	EPSCs [4,55], primed PSCs [28]
Dppa5	F：GATCTCGAATCCCGTATGT<br>R：CAAGTAAGGACCGTAAACCA	104	primed PSCs [28]
Tbx3	F：TGACGGCATACCAGAATGATAAGA<br>R：CTGGGCAAAGCAGTTGAAGG	212	primed PSCs [28,29]
Albumin	F：GCGCTCATAGTTCGTTACAC<br>R：TCAGGACCAGGGACAGATAG	157	endoderm [25,27]
Ncstn	F：CCCAGGATGACAGATAGAAGC<br>R：ACAGGGCAGCACAGAAAGAG	101	endoderm [25,29]
Myh11	F：AAGTTCAAGTCCACCATTTCGG<br>R：CCCTCGCATTTCCTTTCTCC	205	mesoderm [25]
Desmin	F：TGTCCAAGCCAGACCTCACC<br>R：GCCTCATCAGGGAATCGTTAG	249	mesoderm [27]
Nefl	F：AAACGCCGCTATGTGGAGAC	163	ectoderm [25,27]

	R: CGAGACTGGGCATCAAGGAG		
Nestin	F: CCACTCCTCTGGGCTTCTACC<br>R: CCTCATCTCCCTCCTCCTCTG	168	ectoderm [25,51]
Dazl	F: GGGTCGCTTTGCTTATCCGC<br>R: TGCAGCAGACATTACTGCGA	183	late PGC [4,51]
Prdm1	F: CAGTGCCGTGAAGTTTCCA<br>R: AAGGATGCCTCTGCCTGAAC	189	early PGC [52]
Stella	F: CCCGCCTTTCAATCTGTCTCC<br>R: TCGCCGAACCGTGTATCGAA	219	early PGC [51]
Sox17	F: CTGGAGGAGCGGAGCAAATC<br>R: CAGGGCAACTGTGGGAAACC	173	XEN cells [25,81], early PGC [4,52]
Foxa2	F: AAGATGCTGACCCTGAGCGAGAT<br>R: GCGAGTGGCGGATGGAGTT	100	EPSCs [4], primed PSCs [29]
Fgf5	F: CAGCACGTCTCTACCCACTTT<br>R: CTTGACCGTGTTGGGGCTT	159	primed PSCs [29]
Lif	F: TGTCACAGCAACCTCATGAACCAG<br>R: TTCACAGCACCAGGATTGAGGCTC	278	
Lifra	F: GGGTCAATCAGAATCAACAC<br>R: GGTAAATGCCAGGAAAGAGT	120	
Lifrb	F: TGAGATTCCTG ATCCTCAACACAGG<br>R: ATGAGGCAGGGTCCAGACTGAGAT	220	
bFgf	F: AGCACTCCCACCACTACAA<br>R: GAGCCAACGCCTAACAAC	156	
Fgfr1	F: GCGGGTAACTCTATCGGACTC<br>R: TTGGTGCCACTCTTCATCTTG	185	
Fgfr2	F: CTGCCGCCAACACTGTCA<br>R: CGGATGGAACCACGCTTT	165	
Nodal	F: GTCACATAGCCATCTAATCCAA<br>R: CAGACTCCACAGACCCTTCAT	129	
Activin	F: TGCGCATTGACATGTACGCC<br>R: AGCTCCTCCAAGGACGGGTG	143	
Sox7	F: ACAGCCGTCCTTGACTTTCG<br>R: CCAATCCGTCCCTCACTTTA	134	XEN cells [25]
Lama1	F: TGGCAAATCAGAAGAGGAGTC<br>R: CAGAGGGTCACAGTCACAAGG	182	XEN cells [25,81]
Dab2	F: GGACTGGCAAACAGGAAGCG<br>R: TTGTGGAGGTGGGATAATGG	248	XEN cells [25]
Pdgfα	F: TGGACAAGTGAAAGGCAAAG<br>R: CTCGTGGACAGAAATGGTGA	184	XEN cells [25,81]
Apoe	F: GAAGATGAGGGTTCTGTGGGTT<br>R: TGGGTGACCTTGGTGCTGA	228	XEN cells [81]
Gata4	F: ATGCGTCCCATCAAGACAGAGC<br>R: GGTGACTGGCTGACCGAAGAT	173	XEN cells [25,81]

Col4a1	F: CCTCTGGATTGGCTACTCCTTTG<br>R: TGAACATCTCGCTCCGCTCTAT	200	XEN cells [25,81]
Hnf4a	F: CCACAGGCAAACACTACGGG<br>R: TTCTGGACGGCTTCCTTCTTC	196	XEN cells [25,81]
Cdx2	F: AGAACCCCCAGGTCTCTGTCTT<br>R: CAGTCCGAAACACTCCCTCACA	101	TSCs [4,49,55,80]
Gata2	F: CCACTCTGGCTCCCACCTCT<br>R: CTGCCGCTTTCCATCTTCAT	189	TSCs [55]
Gata3	F: CGTCCTGTGCAAACTGTCAA<br>R: CCTTCTTCATGGTCAGGGGT	132	TSCs [4,49,55]
Krt7	F: TCGTGGTGCTGAAGAAGGAT<br>R: CCTTGGACTGCAGCTCTTTC	138	TSCs [4,49,55]
Krt8	F: GGCATCATCGCCGAGGTCA<br>R: ATCTCCGAAATCTCCGTCTT	158	TSCs [80]
Hand1	F: CAAGGCCGAACTCAAGAAGG<br>R: CTGTGCGCCCTTTAATCCTC	119	TSCs [4,49]
Eomes	F: CACCGCCACCAAACCGAGAT<br>R: TTTGCCGCAGGTCACCCACT	177	TSCs [4,55]
Stat3	F: AGAAGGACATCAGCGGTAAGA<br>R: GAGGTAGACCAGCGGAGACA	146	
Gp130	F: CAGTAGTGGTATTGGAGGGT<br>R: CTAACAAGGGCTGGGTGG	191	
Gapdh	F: ACTCACTCTTCTACCTTTGATGCT<br>R: TGTTGCTGTAGGCCAAATTCA	200	

Table S4. CellRanger analysis statistics table of pEPSCs differentiated for 96 hours in LCDM medium (namely pEPSC-LT).

**Sample**	**pEPSC-LT**
Estimated_Number_of_Cells	14,822
Mean_Reads_per_Cell	24,142
Median_Genes_per_Cell	2,595
Number_of_Reads	357,835,034
Valid_Barcodes	97.8
Sequencing_Saturation	46.7
Reads_Mapped_to_Genome	97.8
Reads_Mapped_Confidently_to_Transcriptome	81.3
Fraction_Reads_in_Cells	77.1

Table S5. Full names, abbreviations and proportions of different cell types of eight annotated clusters.

Order	Cell types	Abbreviation	Proportion
**1**	inter cells like PSCs and TSCs	Inter_Cell_1	3226(21.77%)
**2**	other cells	others_1	3078(20.77%)
**3**	XEN-like cells	XEN	2492(16.81%)
**4**	inter cells like TSCs and XEN cells	Inter_Cell_2	1723(11.63%)
**5**	PSC-like cells	PSC_2	1683(11.36%)
**6**	PSC-like cells	PSC_1	1343(9.06%)
**7**	other cells	others_2	1070(7.22%)
**8**	TSC-like cells	TSC	206(1.39%)
