# Supplementary material for: The Trilineage Coexistence Observed During the Differentiation of Porcine EPSCs
Source: Cells. 2026 May 21;15(10):954. doi: 10.3390/cells15100954 (PMC13204623; doi:10.3390/cells15100954)
Supplement: Supplementary file 1 [file cells-15-00954-s001.zip › Supplemental Figures.pdf]

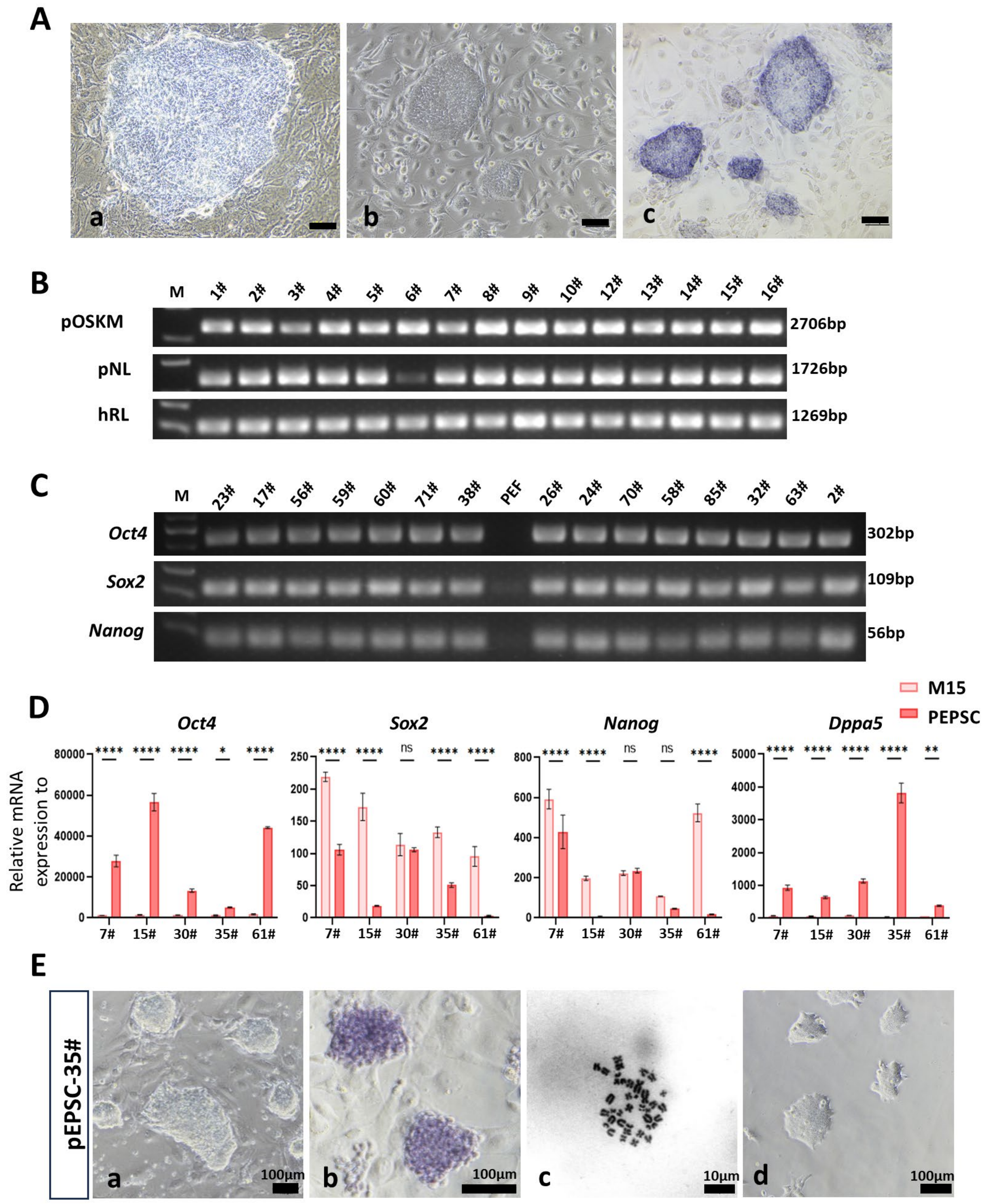

Figure S1. Characterization of colonies cultured in M15 medium and PEPSC medium. (**A**) Morphology and alkaline phosphatase (AP) staining of the colonies. a, Passage 0 colonies; b, Passage 1 colonies; c, AP staining at passage 1. Scale bars, 100 μm. (**B**) PCR analysis of exogenous transcription factors in passage 1 colonies. pOSKM, PB-Tre-pOSKM (porcine *Oct4*, *Sox2*, *Klf4* and *c-Myc*); pNL, PB-Tre-pNhL (porcine *Nanog* and human *LIN28*); hRL, PB-Tre-hRL (human *RARG* and *LRH1*). M: 1kb ladder. (**C**) RT-PCR analysis of endogenous *Oct4*, *Sox2* and *Nanog* expression in passage 1 colonies. M: DL2000 marker. (**D**) RT-qPCR analysis of endogenous *Oct4*, *Sox2*, *Nanog* and *Dppa5* expression in passage 1(M15 medium) and passage 2 (PEPSC medium) colonies. Data are mean ± SD. Statistical significance: *$p < 0.05$, **$p < 0.01$, ****$p < 0.0001$, ns, not significant. (**E**) Morphology, AP staining and karyotype analysis of pEPSC-35#. a, Colony morphology on STO feeders at passages 25; b, AP staining; c, karyotype analysis; d, Feeder free culture at passages 20. Scale bars, 100 μm (a, b, d); 10 μm (c).

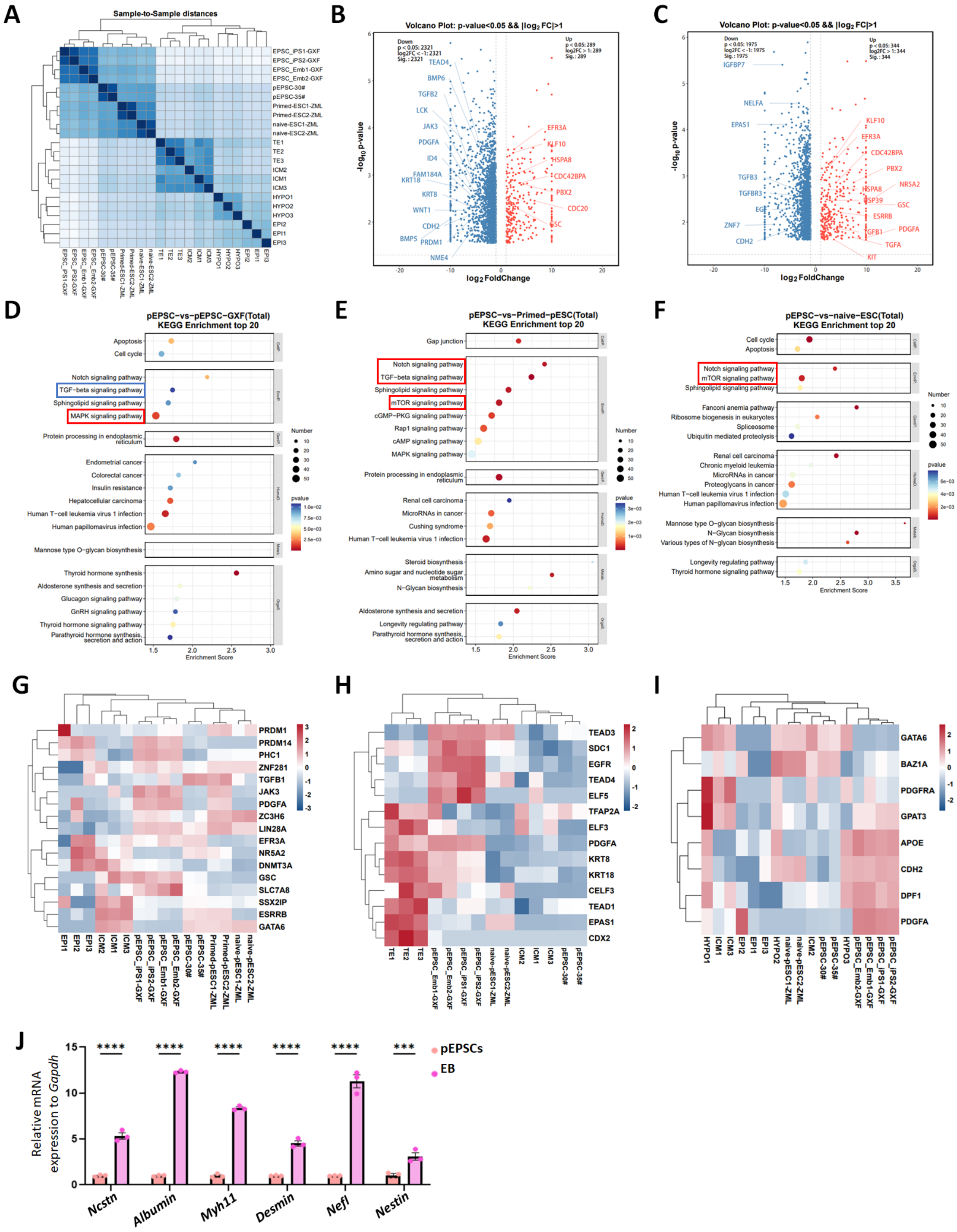

Figure S2. RNA-seq analysis of pEPSCs and gene expression in EB-like from pEPSCs. (**A**) Pearson correlation analysis of global gene expression across different cell types and embryonic lineages. (**B**) Differential gene expression analysis between our pEPSCs and naïve ESCs. (**C**) Differential gene expression analysis between our pEPSCs and primed ESCs. (**D**) KEGG enrichment analysis of our pEPSCs versus porcine EPSC-GXF. (**E**) KEGG enrichment analysis of pEPSCs versus porcine primed ESCs. (**F**) KEGG enrichment analysis of pEPSCs versus porcine naïve ESCs. (**G**) Expression heatmap of core pluripotency genes in our pEPSCs, porcine EPSC-GXF, naïve ESCs, primed ESCs, EPI, and ICM. (**H**) Expression heatmap of TE-associated genes in our pEPSCs, porcine EPSC-GXF, naïve ESCs, and TE and ICM. (**I**) Expression heatmap of HYPO-

associated genes in our pEPSCs, porcine EPSC-GXF, naïve ESCs, and EPI, HYPO and ICM. (**J**) RT-qPCR analysis of differentiation marker expression in EB-like spheroids versus pEPSCs. Data are depicted as mean ± SD. Statistical significance: ***$p < 0.001$, ****$p < 0.0001$.

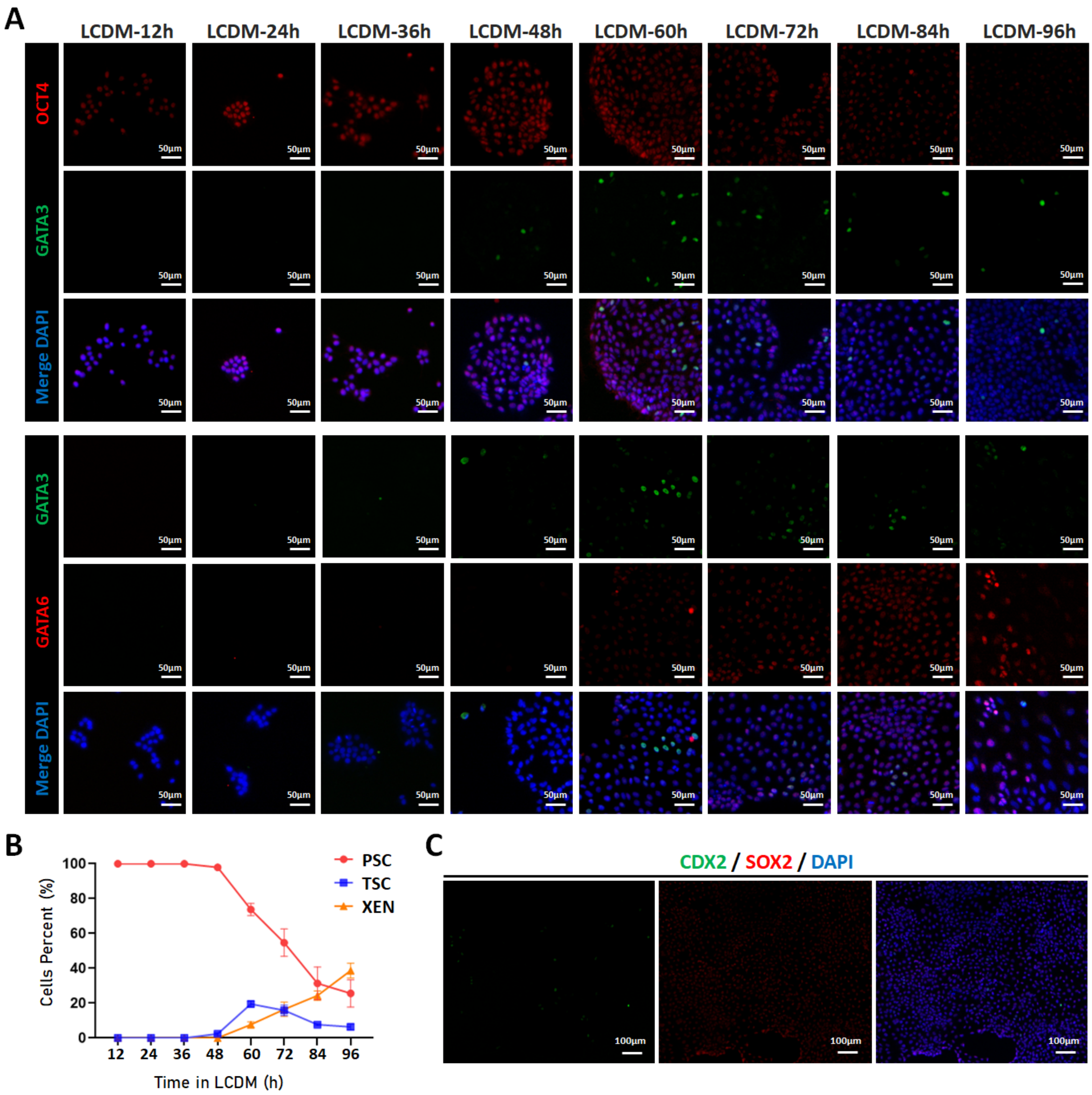

Figure S3. Immunofluorescence characterization of pEPSCs differentiated in LCDM medium. (**A**) Immunofluorescence staining of OCT4, GATA3 and GATA6 in the differentiating pEPSCs in LCDM medium at 12, 24, 36, 48, 72, 84 and 96 hours. Scale bar, 50 μm. (**B**) The temporal evolution of the proportions of PSC ($OCT4^{+}/GATA3^{-}$), TSC ($GATA3^{+}$), and XEN ($GATA3^{-}/GATA6^{high}$) cell populations at 12, 24, 36, 48, 72, 84 and 96 hours based on the immunofluorescence results presented in S3A. Data are mean ± SD. (**C**) Immunofluorescence staining of differentiated cells after two passages in LCDM. Scale bar, 100 μm.

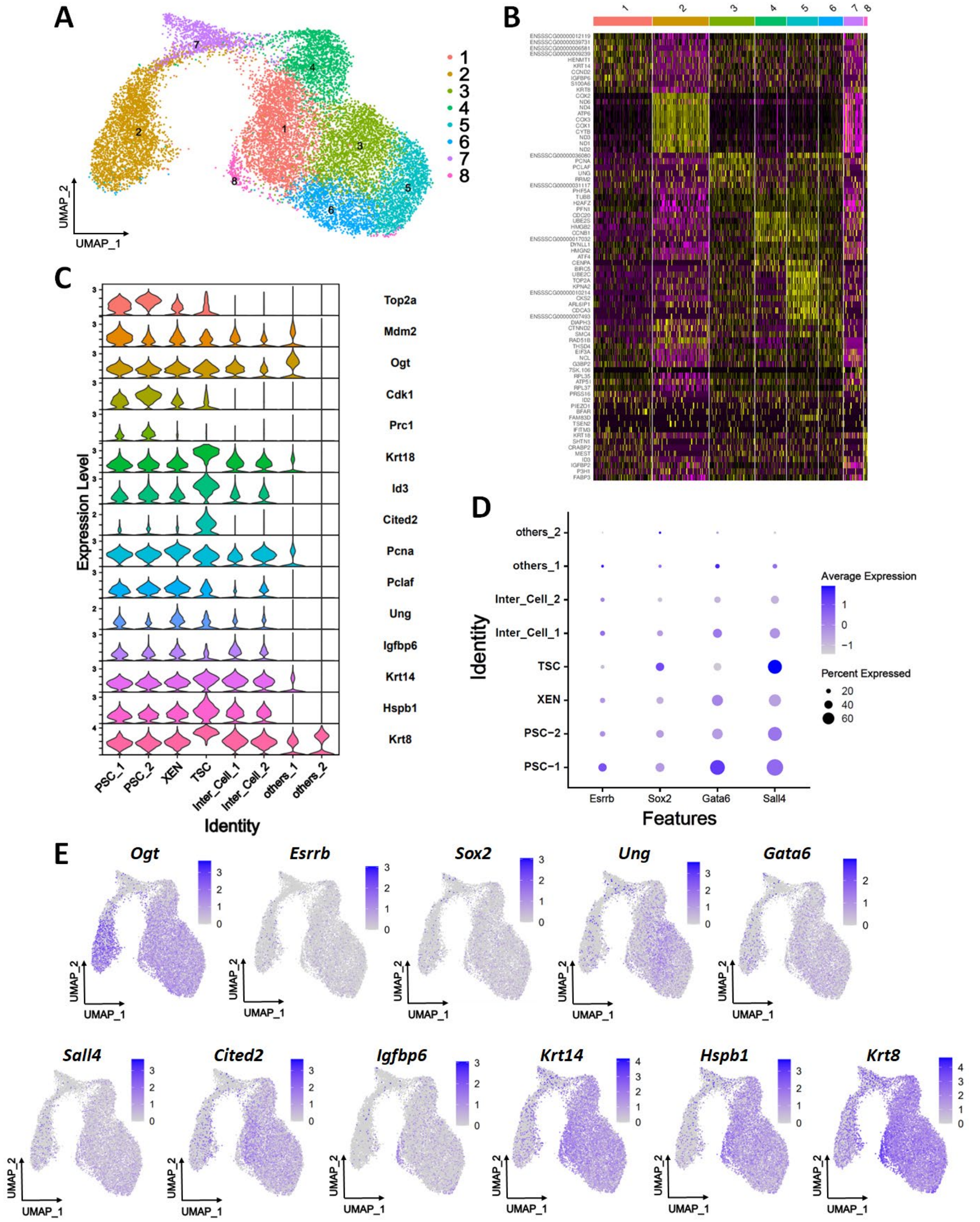

Figure S4. Single-cell transcriptomic analysis of pEPSCs differentiated in LCDM medium. (**A**) Network-based clustering identifying eight cell clusters from transcriptome sequencing data. (**B**) Heatmap showing normalized average expression of the top 10 enriched genes for each cluster. (**C**) Violin plots showing expression levels of marker genes across cell clusters. (**D**) Supplemental dot plot showing the expression level (color scale) and percentage of expressing cells (dot size) for the marker genes across cell types. (**E**) Supplemental UMAP visualization of all cells colored by expression levels of marker genes. Purple to gray: high to low expression.

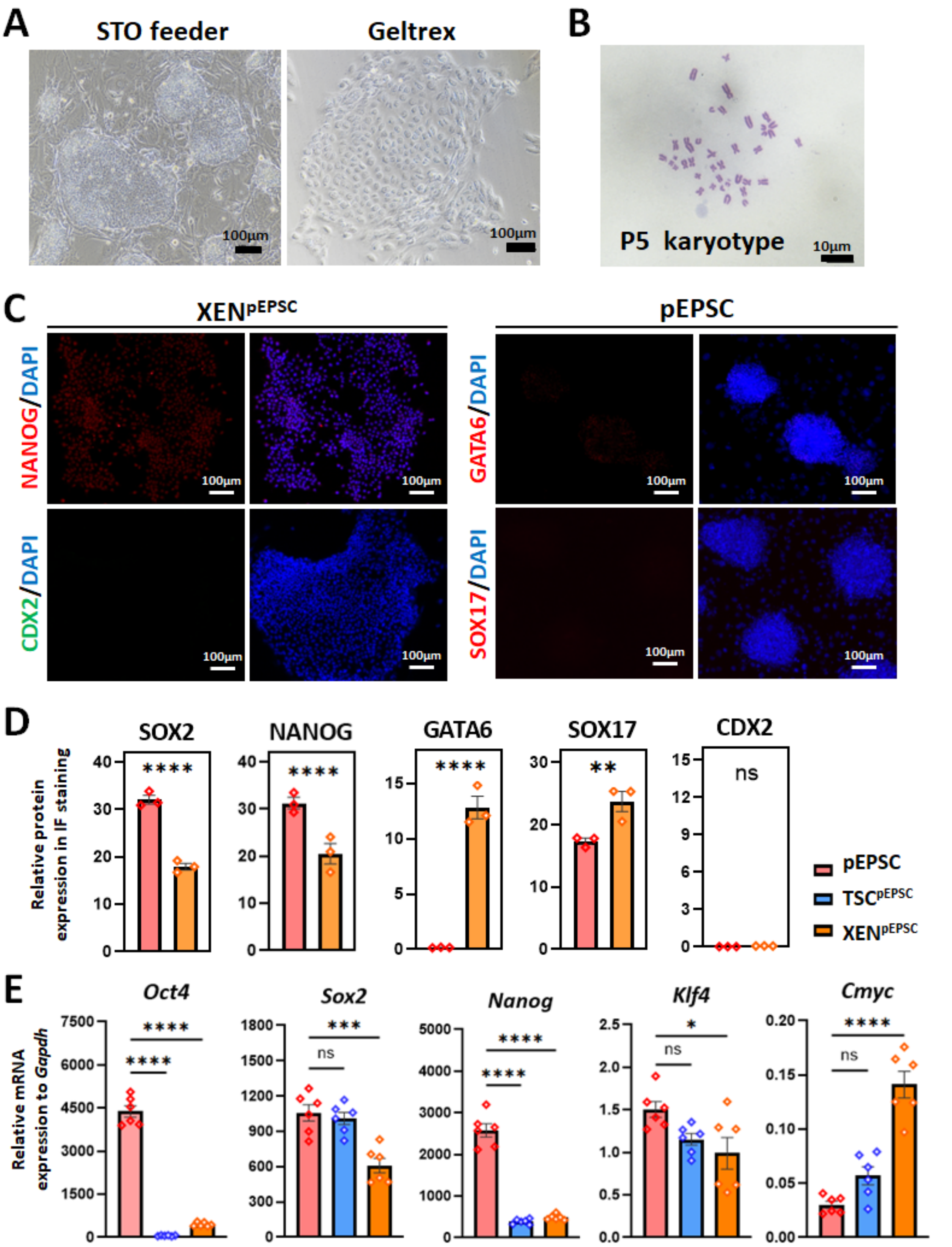

Figure S5. Derivation and characterization of XEN^{pEPSC} cells. (**A**) Morphology of XEN^{pEPSC} cells on STO feeder and Geltrex-coated dishes. Scale bars, 100 μm. (**B**) Karyotype analysis of XEN^{pEPSC} at passage 5. Scale bars, 10 μm. (**C**) Immunofluorescence staining of NANOG, CDX2 in XEN^{pEPSC} colonies, and GATA6 and SOX17 in pEPSCs on Geltrex-coated dishes. Scale bar, 100 μm. (**D**) Quantitative analysis of immunofluorescence signal intensity normalized to DAPI from fig. 5B and fig. S5C. (**E**) RT-qPCR analysis of pluripotency markers (*Oct4*, *Sox2*, *Nanog*, *Klf4*, and *c-Myc*) in pEPSCs, TSC^{pEPSC} and XEN^{pEPSC} cells. Data are mean ± SD. Statistical significance: *$p < 0.05$, **$p < 0.01$, ***$p < 0.001$, ****$p < 0.0001$, ns, not significant.

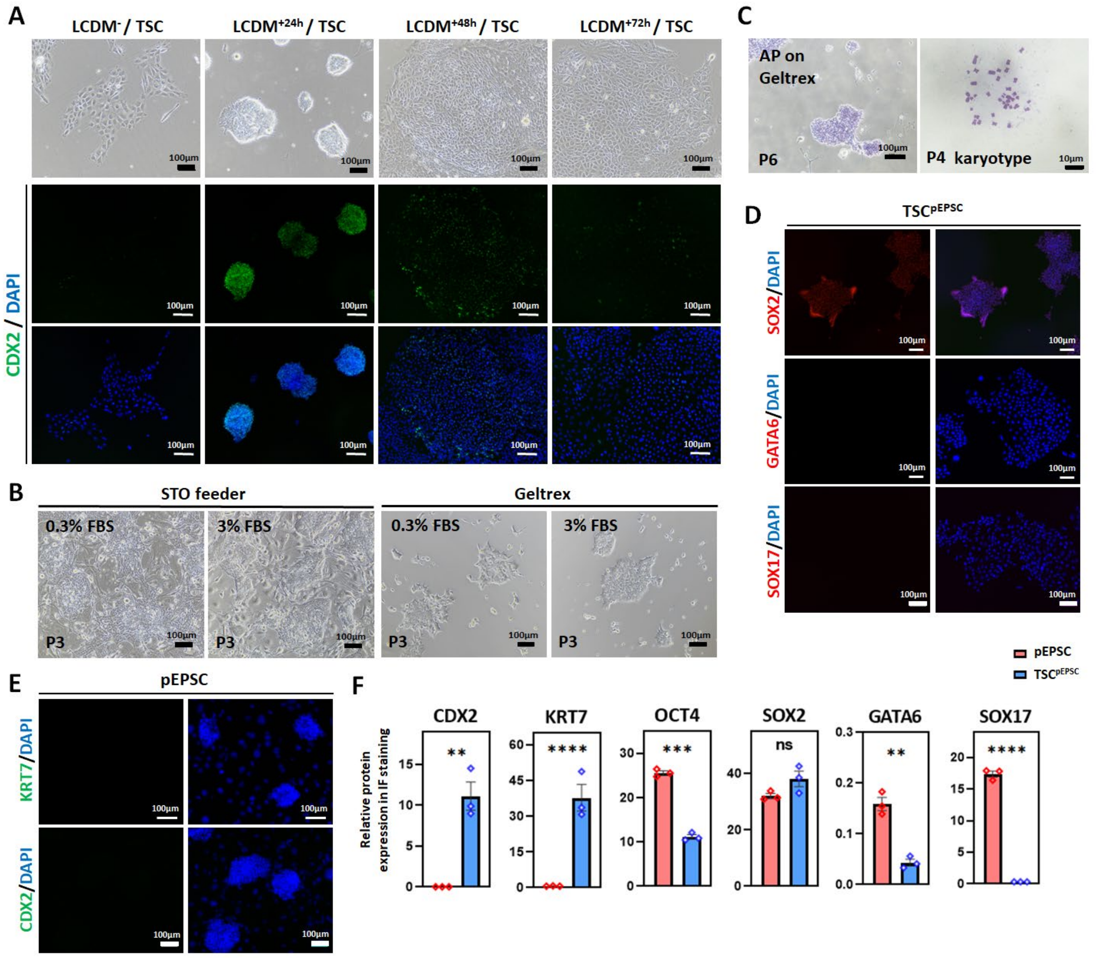

Figure S6. Derivation and characterization of TSC^{pEPSC}. (**A**) Morphology and immunofluorescence staining after switching to TSC culture system at 0 (-), 24, 48, and 72 hours of differentiation in LCDM. Scale bar, 100 μm. (**B**) Morphology of TSC^{pEPSC} cells differentiated from pEPSCs on STO feeder and Geltrex-coated dishes, under 0.3% and 3% FBS at passage 3. Scale bars, 100 μm. (**C**) AP staining (P6) and karyotype analysis (P4) of TSC^{pEPSC}. Scale bars, 100 μm (AP staining); 10 μm (karyotype). (**D**) Immunofluorescence staining of pluripotency marker SOX2 and HYPO lineage markers (GATA6 and SOX17) in TSC^{pEPSC} colonies on Geltrex-coated dishes. (**E**) Immunofluorescence staining of TE lineage markers (KRT7 and CDX2) in pEPSCs. (**F**) Quantitative analysis of immunofluorescence signal intensity normalized to DAPI from fig. 5G and fig. S4D,E. Data are mean ± SD. Statistical significance: $**p < 0.01$, $***p < 0.001$, $****p < 0.0001$, ns, not significant.

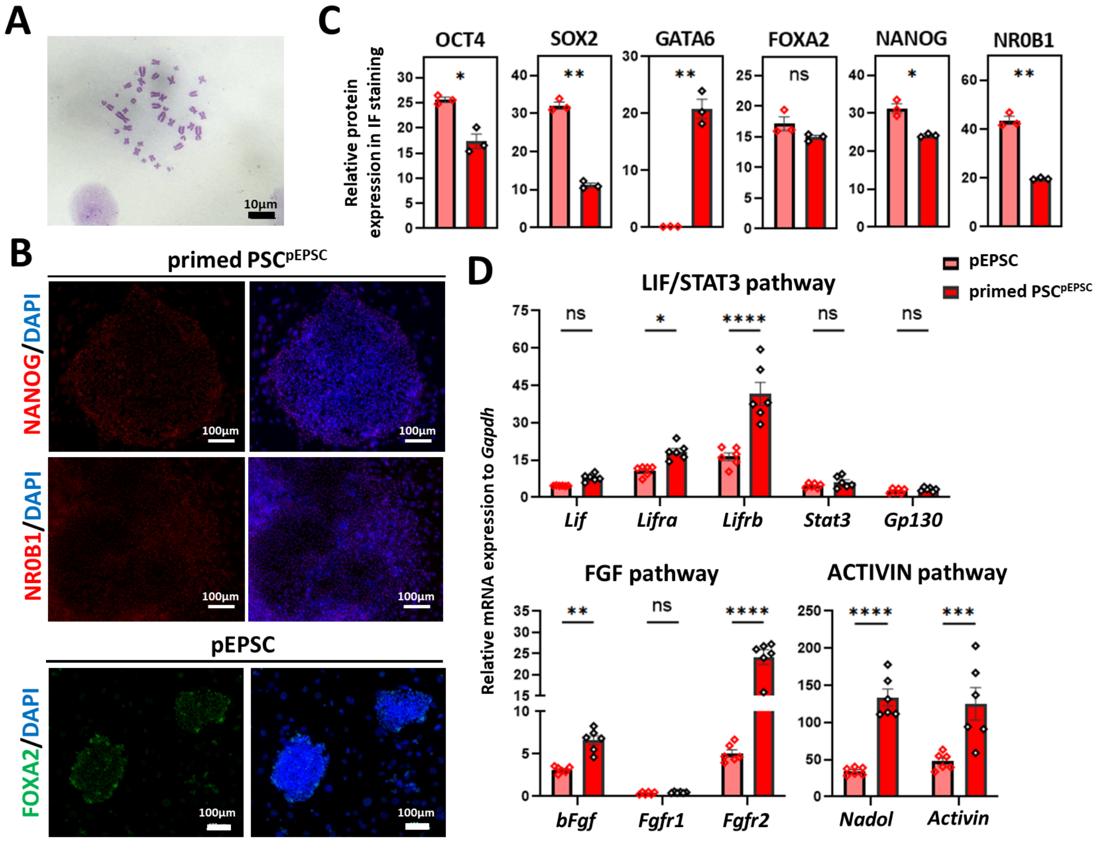

Figure S7. Derivation and characterization of primed PSC^{pEPSC}. (**A**) karyotype analysis of primed PSC^{pEPSC} cells at passage 13. Scale bars, 10 µm. (**B**) Immunofluorescence staining of pluripotency markers NANOG in primed PSC^{pEPSC} and FOXA2 in pEPSC colonies. Scale bar, 100 µm. (**C**) Quantitative analysis of immunofluorescence signal intensity normalized to DAPI from fig. 6B and fig. S6B. (**D**) RT-qPCR analysis of LIF/STAT3, FGF, and ACTIVIN pathway gene expression in pEPSCs and primed PSC^{pEPSC} lines. Data are mean ± SD. Statistical significance: $*p < 0.05$, $**p < 0.01$, $***p < 0.001$, $****p < 0.0001$, ns, not significant.
